# Nickel stress-tolerance in plant-bacterial associations

**DOI:** 10.7717/peerj.12230

**Published:** 2021-09-29

**Authors:** Veronika Pishchik, Galina Mirskaya, Elena Chizhevskaya, Vladimir Chebotar, Debasis Chakrabarty

**Affiliations:** 1 All-Russia Research Institute for Agricultural Microbiology, Saint-Petersburg, Pushkin, Russian Federation; 2 Agrophysical Scientific Research Institute, Saint-Petersburg, Russian Federation; 3 CSIR-National Botanical Research Institute, Lucknow, India

**Keywords:** Nickel stress, plant defense system, plant-bacterial associations

## Abstract

Nickel (Ni) is an essential element for plant growth and is a constituent of several metalloenzymes, such as urease, Ni-Fe hydrogenase, Ni-superoxide dismutase. However, in high concentrations, Ni is toxic and hazardous to plants, humans and animals. High levels of Ni inhibit plant germination, reduce chlorophyll content, and cause osmotic imbalance and oxidative stress. Sustainable plant-bacterial native associations are formed under Ni-stress, such as Ni hyperaccumulator plants and rhizobacteria showed tolerance to high levels of Ni. Both partners (plants and bacteria) are capable to reduce the Ni toxicity and developed different mechanisms and strategies which they manifest in plant-bacterial associations. In addition to physical barriers, such as plants cell walls, thick cuticles and trichomes, which reduce the elevated levels of Ni entrance, plants are mitigating the Ni toxicity using their own antioxidant defense mechanisms including enzymes and other antioxidants. Bacteria in its turn effectively protect plants from Ni stress and can be used in phytoremediation. PGPR (plant growth promotion rhizobacteria) possess various mechanisms of biological protection of plants at both whole population and single cell levels. In this review, we highlighted the current understanding of the bacterial induced protective mechanisms in plant-bacterial associations under Ni stress.

## Introduction

Heavy metals (HM) contaminant of agricultural land and water causes major environmental and human health problems (Roy & McDonald, 2015; Ihedioha, Ukoha & Ekere, 2017). Nickel (Ni) has been indicated as one of the most dangerous HM for the environment, and Ni affected plants undergo a severe stress condition (Hussain et al., 2013; Pietrini et al., 2015).

Ni enters the soil through a variety of sources such as metal smelters, industrial effluents, Ni- oxide nanoparticles during the manufacture of electronic devices and catalysts, wastewater, including uses of fertilizers and pesticides (Song et al., 2008; Cabanillas et al., 2012). Among heavy metals, Ni is characterized by barrier-free penetration into the aboveground organs of plants (Fabiano et al., 2015; Deng et al., 2018). Ni induces cytotoxic and genotoxic effects on plants (Magaye & Zhao, 2012; Manna & Bandyopadhyay, 2017). Excessive accumulation of Ni in plants leads to oxidative stress, accompanied by an increase in the accumulation of ROS (reactive oxygen species) (Sharma & Dietz, 2009), an inhibitor of growth, mineral nutrition (Parida, Chhibba & Nayyar, 2003), photosynthesis (Prasad, Dwivedi & Zeeshan, 2005), membrane functions, carbohydrate metabolism (Seregin & Kozhevnikova, 2006) and water regime of plants (Llamas & Sanz, 2008).

Plants have a number of potential mechanisms to protect against high concentrations of heavy metals, with which they manage to survive under metal stress (Schützendübel & Polle, 2002). Resistance to heavy metal toxicity depends on reduced absorption, increased in vacuolar sequestration and enhanced expression of defense proteins. There are some recent reviews about strategies adapted by plants to neutralize and overcome the Ni stress (Fabiano et al., 2015; Sachan & Lal, 2017; Shahzad et al., 2018b; Deng et al., 2018) that indicate a great interest among scientific community.

PGPR (Plant Growth Promotion Rhizobacteria), inhabiting plant rhizosphere and rhizoplane and interacting with root exudates and soil microbial communities, form strong associations with plants (Brencic & Winans, 2005). PGPR in native ecosystems play a key role in protecting plants from various stress factors, including high concentrations of HM. Under HM stress sustainable plant-microbial associations are formed for the joint survival of both partners (Jing, He & Yang, 2007). Such plant-bacterial associations were described for plants: Ni-hyperaccumulators *Alyssum bertolonii* (Mengoni et al., 2001) and *Tlaspi goesingense* (Idris et al., 2004) with their Ni-resistant dominant bacteria from genera *Pseudomonas, Methylobacterium, Rhodococcus* and *Okibacterium*. Also, such associations are artificially formed when PGPR are used for phytoremediation, the success of which depends on the correctly used association. Endophytic bacteria penetrate into the root cortex, live inside the plant roots in the root cells, improved nutrient uptake and plant growth. Endophytic bacteria are also successfully used for phytoremediation (Jan et al., 2019; Naveed et al., 2020). Phytoremediation is a green strategy that uses hyper-accumulator plants and their rhizospheric microorganisms to stabilize, transfer or degrade pollutants in soil, water and environment. This technology is considered as well-efficient, cheap and adaptable with the environment (Nedjimi, 2021).

This review focuses on plant defense mechanisms in plant-bacterial associations under Ni stress. Previous reviews touched this topic a little (Pishchik et al., 2016; Egamberdieva, Abd-Allah & Silva, 2016), focusing mainly on other heavy metals, specifically on Pb, Cd, and Zn.

## Survey methodology

Initially we analyzed the closed-relation reviews to our topic, to choose the general direction and novelties of our review. The key words for each section of review were selected and used in search of Web of Science and Google Scholar databases. To collect all relevant information we used the following keyword combinations: nickel (Ni) contamination and soil; Ni stress and plants; Ni and physiological destructions in plants; Ni stress and plant antioxidant enzymes; Ni and phytochelatins, Ni and metallothioneins; Ni and proline; Ni and salicylic acid; Ni and phytohormones; Ni- stress and plant genes; Ni-stress and bacterial genes; Ni- stress and microbial community; Ni- stress and PGPR; Ni-stress and endophytic bacteria; Ni stress and plant—bacterial association; Ni and bioremediation. The abstracts of selected papers were initially screened according to plan of our review, which were focused on topics of survival of plant- bacterial systems under Ni stress. We have mostly focused on the papers of 2000–2020, since the main research in this area were done at this time.

### Ni in soil, in plants and in bacterial cells

The contamination of soil with Ni and other HM resulting due to wastes from heavy industry and nonferrous metallurgy is a major environmental concern. However, Ni can be introduced into the environment with mineral fertilizers, waste water, sludge, oil spills and household rubbish (Ghosh & Singh, 2005; Kozlov, 2005; Vodyanitskii et al., 2011). The worldwide average concentration of Ni in natural soils is 22 mg kg^−1^ (Kabata-Pendias, 2000). The range of nickel (Ni) concentrations may reach 200–26,000 mg kg^−^^1^ in polluted soils, as compared with natural soils (10–1,000 mg kg^−^^1^) (Sreekanth et al., 2013; Yusuf et al., 2011). The maximum recorded nickel concentration in contaminated soils was observed in Canada and reached 26,000 mg kg^−1^ (Kabata-Pendias, Mukherjee & Arun, 2007). The content of Ni in polluted soils of the city exceeds Tentative Permissible Concentrations level by 10–86 folds (Vodyanitskii et al., 2011) and 75 times higher with severe pollution compared to background (Evdokimova, Kalabin & Mozgova, 2011).

Ni is the main pollutant of farmlands in south and central China (Rizwan et al., 2017), the Ni concentrations in China soils may increase in 6.5 times compared to background (Ding et al., 2008). Soils of agricultural land near industrial areas in India contain 47 to 178 mg kg^−1^ Ni (Rajindiran et al., 2015). In this regard, the problem of the accumulation of excess Ni in agricultural products arises.

Ni is an essential trace element (in low concentrations 0.01–5 µg/g dry weight) for plants. Ni (II) is a functional component in urease (Gerendas, Zhu & Sattelmacher, 1998), glyoxalases, peptide deformylases, methyl Co-M reductases, hydrogenases and superoxide dismutases (Aziz, Gad & Badran, 2007). However the high level of Ni concentrations (more than 10^−4^ M/l) can lead to toxicity symptoms and growth inhibition in most plants (Hall, 2002). The average Ni content in wheat leaves (plant-excluder, accumulates metals in roots) is 0.34 mg kg^−1^ (Kabata-Pendias, Mukherjee & Arun, 2007). The family *Brassicaceae* has the highest number of hyper accumulator plants (Reeves & Baker, 2000). Such representatives of the family as *Alyssum caricum* (Dudley) and *Thlaspi oxyceras* (Boiss.) can accumulate significant amounts of Ni (up to 12,273 and 13,778 mg/kg, respectively) in the leaves (Shahzad et al., 2018b).

Ni is an essential component of bacterial enzymes which catalyze metabolic reactions with molecular hydrogen, nitrogen, carbon monoxide and carbon dioxide and is involved in pathogenesis and detoxifications processes (Mulrooney & Hausinger, 2003). However, influence of high concentrations of Ni in bacterial cells leads to oxidative stress resulting in damage of nucleic acids, lipid peroxidations, and enzymes inactivation (Eitinger et al., 2005).

### Physiological and biochemical destructions caused by Ni in plants

Metal toxicity leads to molecular changes in plants such as: (a) formation of reactive oxygen species (ROS) by auto-oxidation and Fenton reaction (Noctor, Reichhel & Foyer, 2018); (b) locking of main functional groups in biomolecules; (c) expulsion of main metal ions from biomolecules (Gajewska & Skłodowska, 2007). Ni ions in high concentrations have a destructive effect on growth, mineral nutrition, photosynthesis (Zaid et al., 2019) carbohydrate transport and water relations (Seregin & Kozhevnikova, 2006). The increasing levels of Ni stress enhanced methylglyoxal, electrolyte leakage, hydrogen peroxide, and lipid peroxidation content in plants (Zaid et al., 2019). Ni decreases seed germination and seedling growth due to change in the activity of hydrolytic enzymes, followed by a delay in the transportation of mobilized reserves from endosperm to the embryonic axis (Ashraf et al., 2011). Ni may disrupt the membrane stability (Shahzad et al., 2018b) by reducing the uptake of Ca and Zn (Taiz & Zeiger, 2006). High concentration of Ni in plants leads to mitotic abnormalities, chromosomal aberrations and decrease in the rate of cell stretching (Sreekanth et al., 2013; Manna & Bandyopadhyay, 2017).

It has been reported that Ni stress can reduce cytosine methylation levels in clover and hemp and the decrease in methylation depends upon the dosage of the heavy-metal stress. Methylation-sensitive amplification polymorphism (MSAP) data shows that the methylation patterns of different plants within the CCGG sites are similar before and after HM stress, suggesting that the stress-induced changes in methylation are not distributed randomly (Aina et al., 2004).

The differential regulation of chloroplastic heat shock protein (Cp-sHSPs or HSP26.13p) in *Chenopodium album* protects the plant both from heat and Ni as well as other (Cu and Cd) HM stresses (Haq et al., 2013). It was revealed in proteome analysis of different plant species that ubiquitin activity can be reduced significantly by Ni and other HM (Cd, Pb, Co, Cu, Cr, Hg) at 100 µM concentration, whereas low concentrations can induce 26S proteasome activity. Although these metals induce the accumulation of ubiquitin conjugated proteins, the abundance of 20S core protein in UPS system is not changed (Aina et al., 2007; Pena et al., 2008).

### Plants defense system

Plants have different levels of protection against elevated levels of HM. The first level is the physical barrier, represented by various morphological structures, such as a thick cuticle, cell walls, trichomes (Hall, 2002; Fourati et al., 2016). Trichomes can secrete various secondary metabolites to detoxify heavy metals (Hauser, 2014). High concentrations of Ni absorbed into the vacuole, which protects the cytoplasm from the toxic effect (Krämer et al., 2000). The sequestration of Ni into the leaf vacuole can be connected with a vacuolar metal-ion transporter protein (TgMTP1) (Persans, Nieman & Salt, 2001). The second level of biochemical protection is the inclusion of heavy metals into plant tissue. In this case, the plant synthesizes various substances of an enzymatic and non-enzymatic nature (Manoj et al., 2020). The response of plants to Ni stress depends on the plant species. At the same time, intraspecific and interspecific hybrid differences in the presence of Ni in high concentrations are noted (Amjad, 2020; Amjad et al., 2020).

Under stress of high Ni concentrations, plants trigger numerous adaptive mechanisms to neutralize its action, including the induction of many low molecular weight protein chelators, such as phytochelatins and metallothioneins, specific amino acids, such as proline, and activation of antioxidant enzymes (Dalvi & Bhalerao, 2013; Viehweger, 2014). The equilibrium between the synthesis and detoxification of free radicals in plants is supported by plant enzymes and antioxidants of nonenzymatic nature, such as ascorbate, glutathione, tocopherol, carotenoids and phenols (Mittler et al., 2004).

Glutathione (GSH) plays a significant role in cellular redox balance by binding to Ni and other HM. It was found that elevated GSH concentration driven by constitutively elevated SAT (serine acetyltransferase activity) correlated with increased resistance to Ni stress in *Thlaspi goesingense* (Freeman et al., 2004). Besides, it was shown that plants resistance to HM is clearly linked to the efficiency of glutathione S-transferases (GST) in the detoxification process (Wu et al., 2019). So, for Ni a negative correlation between GST/peroxydase activities and chlorophyll (Chl) content has been indicated (Helaoui et al., 2020).

Plants synthesize proline in response to nickel stress. That was shown for various plant species, such as *Triticum aestivum* (Gajewska & Skłodowska, 2009), *Brassica oleracea var. capitata* (Pandey & Sharma, 2002), *Pisum sativum* (Gajewska & Skłodowska, 2005). Proline functioning as osmolyte is also a defense against Ni toxicity (Seregin & Kozhevnikova, 2006).

Proline level was higher in Ni-treated rice plants compared to Cd-treated plants. However, Ni cations in a high concentration (1.0 mM) significantly decreased proline synthesis (Jan et al., 2019).

Plants can decrease Ni toxicity, chelating Ni cations with various organic acids. Malic acid synthesis is associated with Ni tolerance in plants such as Ni-hiperaccumulator *Stackhousia tryonii* Bailey (Bhatia, Walsh & Baker, 2005), ryegrass, and maize (Yang et al., 1997).

Ni-tolerance of the eight different species (*Homalium kanaliense* (Vieill.) Briq., *Casearia silvana* Schltr, *Geissoishirsuta* Brongn. & Gris, *Hybanthus austrocaledonicus* Seem, *Pycnandra acuminata* (Pierre ex Baill.) Swenson & Munzinger (syn *Sebertia acuminata* Pierre ex Baill.), *Geissois pruinosa* Brongn & Gris, *Homalium deplanchei* (Viell) Warb. and *Geissois bradfordii* (H.C. Hopkins) was associated with citric acid (Callahan et al., 2012).

***Antioxidant enzymes.*** The antioxidant enzymes: superoxide dismutase (SOD), catalase (CAT), glutathione peroxidase (GSH-Px), guaiacol peroxidase(GPX), peroxiredoxins (Prxs) and enzymes of the ascorbate-glutathione (AsAGSH) cycle, such as ascorbate peroxidase (APX), monodehydroascorbate reductase (MDHAR), dehydroascorbate reductase (DHAR), and glutathione reductase (GR) are indicative enzymes for a high level of abiotic stress in plants (Mittler et al., 2004).

The effect of Ni on antioxidant enzymes is different for different types of plants. Pandey & Sharma (2002) reported that Ni reduced CAT and POD activities in cabbage leaves. However in pigeon pea (*Cajanu scajan L.*) there was no change in CAT activity under Ni stress, while SOD, glutathione reductase (GR) and POD activities were increased (Rao & Sresty, 2000). Ni stress significantly decreased activities of CAT and SOD and increased activities of glutathione peroxidase (GSH-Px) in wheat plants (Gajewska et al., 2006; Gajewska & Skłodowska, 2007). In shoots of Ni-stressed *Solanum nigrum* L., an enhanced activity of SOD and APX, accompanied by a decline of CAT activity were observed. In roots, increases in SOD and CAT activities were detected in response to Ni, whilst APX was not increased (Soares et al., 2016).

Data on plants antioxidant activities are summarized in Table 1. Since various actions of Ni on plant antioxidant enzymes are described, further study of this direction is necessary.

**Table 1:** Plants defense mechanisms under Ni stress.

**Plant species**	**Ni conc.**	**Mechanisms**	**Reference**
*Triticum aestivum* L.	25–50 µg/L	(+) SOD activity, proline content	Gajewska et al. (2006), Parlak (2016)
	200 µM	(+) proline content in shoots; POD, GST activities (-) SOD, CAT activities	
*Atropa belladonna* L.	50–200 µM	(+) proline, spermine, spermidine contents (-) content of putrescine	Stetsenko, Shevyakova & Kuznetsov (2011)
*Solanum nigrum* L	100 µM	(+) SOD and CAT activities in roots (+) SOD and APX activities in shoots (-) CAT activity in shoots	Soares et al. (2016)
*Oryza sativa* L.	10–50 µM	(-) MDA concentrations	Rizwan et al. (2017)
	100–200 µM	(+) proline content; POD and CAT activities in roots and shoots (-) SOD activity in roots and shoots	
*Lactuca sativa* L.	400–600 mg/kg of soil	(+) CAT, POD, SOD activities in shoots (+) MDA and GST levels	Zhao et al. (2019)
*Alyssum inflatum* Nyár.	100–400 µM	(+) proline content; (+) SOD, POD, CAT, APX activities	Najafi, Karimi & Ghasempour (2019)
*Hydrilla verticillata* (Lf) Royle	5–15 µM	(+) SOD and CAT activities in leaves and stems; POD activity in leaves (-) POD activity in stems	Song et al. (2018), Zhang et al. (2020)
	20–40 µM	(-) SOD and CAT activities in leaves and stems; POD activity in leaves (+) POD activity in stems	
*Vigna mungo* L.	10–100 µM	(+) proline content	Singh et al. (2012)
*V. cylindrical* L., *V. radiate* L.	50–150 M	(+) SOD, CAT and POD activities in roots	Mahmood et al. (2016)
*Populus nigra* L.	200–800 M	(+) CAT and APX activities in leaves	Kulac et al. (2018)
*Pisum sativum* L*.*	100 M	(+) SOD, POD, CAT, APX, GSH-Px, GR activities, proline, glycinebetaine contents	Balal et al. (2016)
*Landoltia punctate*	0.01–0.5 mg/L	(+) SOD, POD, CAT activities	Guo et al. (2017)
	5–10 mg/L	(-) SOD, POD, CAT activities	
*Grewia asiatica* L.	20 mg/kg of soil	(-) SOD, CAT, POD activities	Zahra et al. (2018)
	40–60 mg/kg of soil	(+) POD activity (-) SOD, CAT activities	
*Glycine max* L.	0.05–20 µM	(+) SOD, POD activities	Reis et al. (2017)
*Catharanthus roseus* L.	2.5–50 mM	(+) proline content, CAT activity	Arefifard, Mahdieh & Amirjani (2014)
*Medicago sativa* L.	50–500 mg/kg	(+) POD activity	Helaoui et al. (2020)
*Avéna sativa* L., *Panicum miliaceum* L.	10–40 ppm	(+) proline content, POD and SOD activities in roots and shoots (-) CAT activity in roots and shoots	Gupta et al. (2017)
*Amaranthus paniculatus* L.	25–150 µM	(+) GSH-Px, SOD activities in leaves (-) APX, CAT, GSH-Px, SOD activities in roots	Pietrini et al. (2015)
*Brassica juncea* L.	100–400 µM	(+) proline content, SOD activity (-) APX, CAT activities	Thakur & Sharma (2016)

**Notes.**

(+) increased (-) decreased APX ascorbate peroxidase CAT catalase MDA malone dialdehyde GSH-Px glutathione peroxidase GST glutathione S-transferase activity GR glutathione reductase POD peroxidase activity SOD superoxide dismutase

***Phytochelatines* (PCs)** are the most important metal-binding ligands, since it is believed that the synthesis of these compounds is one of the key detoxification mechanisms (Chen et al., 2008). PCs are low molecular weight, short-chain thiol repeating proteins that have high affinity for binding to HMs when they are at toxic levels (Lee et al., 2002; Chen et al., 2008; Shukla et al., 2013). PCs are produced in plants from sulfur-rich glutathione (GSH) using phytochelatin synthase (PCS). PCs form high-molecular complexes with toxic metals, including Ni, in the cytosol and subsequently transfer them to plant vacuoles (Song et al., 2014). Induction of PCs synthesis occurs within cells as a result of exposure to various levels of Ni in both the roots and above-ground organs. Nickel accumulation resulted in formation of PCs in *Nicotiana tabacum* L and *Thlaspi japonicum* (Nakazawa et al., 2001; Mizuno et al., 2003).

PCs’ synthesis is considered as one of the protective functions of plants against the stress of nickel and other metals (Talebi et al., 2019). It has been suggested, that PCs may serve as a biological marker for Ni accumulation in plants (Ameen et al., 2019). That suggestion is confirmed in the study of phytochelatins gene expression in response to the action of different concentrations of nickel in alfalfa plants (Helaoui et al., 2020).

Though Ni was a relatively effective activator of PC synthase during *in vitro* studies, a functioning more effective and alternative detoxification mechanisms, such as metallothioneins and histidine in plants was proposed (Cobbett, 2000).

***Metallothioneins (*MTs)** are low molecular weight cysteine-rich proteins (4 –8 kDa) that make up an extremely heterogeneous family of metal-binding proteins that are ubiquitous in cells (Peroza & Freisinger, 2007). In plants, MTs are involved in neutralizing HM toxicity through cell sequestration, homeostasis of intracellular metal ions, and regulation of metal transport (Guo et al., 2013). MTs form metal-thiolate complexes; therefore, they can tolerate to elevated concentrations of metals (Kumar et al., 2012; Mirza et al., 2014).

Ni increases the MTs expressions in *Solanum nigrum* (Ferraz et al., 2012) and *Lupinus luteus* (Jaskulak et al., 2019), that results prove the involvement of MTs in Ni homeostasis and detoxification.

It is shown that MTs genes can be used to create HM-resistant plant-microbial systems and their subsequent application in phytoremediation or phytostabilization technologies (Pérez-Palacios et al., 2017; Tsyganov et al., 2020).

***Phytohormones*** are classified into different groups (auxins, cytokinins, gibberellins, brassinosteroids, salicylic acid, abscisic acid, and jasmonates) and plays different roles in plant growth and development.

A positive non-significant effect of combined application of gibberellins and cytokinins effect on Ni phytoextraction efficiency of *Alyssum corsicum* was demonstrated (Cabello-Conejo et al., 2013). Auxins were found as the most effective phytohormones for increasing Ni yield from Ni hyperaccumulating *Alissum* and *Noccaea* species. All the phytohormones increased plants biomass, but not in all cases the increase in biomass was associated with an increase in nickel yield (Cabello-Conejo, Prieto-Fernández & Kidd, 2014).

It was found that application of gibberellins, cytokinins and auxins generally led to a reduction in shoot Ni concentration of *Alissum* and *Noccaea* species (Cabello-Conejo, Prieto-Fernández & Kidd, 2014).

The application of epibrassinolide (EBL) recovered the growth *Brassica juncea* and reduced Ni uptake in roots and shoots and improved activities of SOD, CAT, APOX and POD (Kanwar et al., 2013), as well as photosynthetic pigments, osmolyte accumulation in *Solanum nigrum* (Soares et al., 2016). Application of EBL under Ni stress helps to obtain large plant biomass but possible mechanism of epibrassinolide is still poorly understood (Shahzad et al., 2018a).

Abscisic acid (ABA) induces ethylene biosynthesis in adult plants and promotes their senescence and abscission (Liu et al., 2016).Under stress conditions, ABA signaling interacts with plants gibberellin and auxin signaling pathways and controls lateral root development (Zhao et al., 2014). Ni stress in rice increased the ABA level and ABA was increased with increased heavy metal concentration (Jan et al., 2019). Opposite, concentration of salicylic acid (SA) decreased significantly under HM stress, which confirmed the antagonistic effect between SA and ABA (Jan et al., 2019).

SA is plant phenolic, and is present in plants as a free and conjugated form (Maruri-López et al., 2019). SA can alleviate HM toxicity, decrease ROS, protect membrane stability, interact with other plant hormones, up-regulate hemeoxygenase, improve the performance of the photosynthetic machinery (Sharma et al., 2020). SA plays a key role in the regulation of plant growth, development, in defense from HM stress and in plant responses (Freeman et al., 2005; Pasternak et al., 2019). It is known, that GSH- Glutathione mediated Ni tolerance mechanism in *Thlaspi* hyperaccumulators is signaled by the constitutively elevated levels of salicylic acid (SA) (Freeman et al., 2005). SA alleviates metal toxicity influencing their uptake and accumulation in plant organs (Dalvi & Bhalerao, 2013). Application of SA under Ni-stress reduced ROS, H_2_O_2_ and MDA contents and lipoxygenase activity, thus up-regulating the capacity of antioxidant defense system in chloroplasts of maize (Wang et al., 2009) and wheat (Siddiqui et al., 2013), accelerated the restoration of growth processes and improves the total alkaloid content in periwinkle (*Catharanthus roseus* L.) (Idrees et al., 2013).

The role of phytohormones in HM stress is discussed in scientific literature but the effects of phytohormones on plants differ and depend on the application rates and time, as well as on the environmental factors and plant species.

### Genes involved in plant protection system to Ni stress

Ni uptake by plant roots can be connected with Fe transporter(s). For instance, in the Ni hyperaccumulator *Alyssum inflatum*, Fe accumulation in roots was stimulated by increased Ni concentrations (Ghasemi, Ghaderian & Krämer, 2009) because of the lack of substrate specificity of AtIRT1. Ni cations could be absorbed *via* the ferrous transporter IRT1 in *A. thaliana* (Nishida et al., 2011; Nishida, Aisu & Mizuno, 2012).

No specific Ni efflux transporter has been identified. When getting into xylem vessels, Ni transport is mainly driven by leaf transpiration (Centofanti et al., 2012). Ni absorption by leaf cells may involve transporters from the ZIP family (ZNT1 and ZNT2), as the gene expression of these transporters triggered under Ni stress (Visioli, Gulli & Marmiroli, 2014).

Based on recent genetic studies, the following genes were proposed as candidates for Ni-stress in different plant defense system: serine acetyltransferase (SAT), glutathione reductase (GR) in *Thlaspi goesingense* (Freeman et al., 2004), glutathione S–transferase in *Betula papyrifera* (Theriault, Michael & Nkongolo, 2016), 1-aminocyclopropane-1-carboxylic acid deaminase (ACC) in *Brassica napus* (Stearns et al., 2005) and in *Quercusrubra* (Djeukam & Nkongolo, 2018), nicotianamine synthase (NAS3) in *Thlaspi caerulescences* (Mari et al., 2006) and *Populus tremuloides* (Czajka, Michael & Nkongolo, 2018), thioredoxin family protein in *Chlamydomonas reinhardtii* (Lemaire et al., 2004) and in *Betula papyrifera* (Theriault, Michael & Nkongolo, 2016)

In recent years, the search for genes responsible for plant resistance to nickel stress became one of the important areas. Several candidate genes that are involved in plant protection against Ni stress have been identified. However, work in this direction should be continued.

There are also very few works concerning the study of the level of expression of plant genes under nickel stress. The main nickel resistance mechanism in *Betula papyrifera* is a downregulation of genes associated with translation (in ribosome), binding, and transporter activities (Theriault, Michael & Nkongolo, 2016). Four nicotianamine synthase genes in *Arabidopsis* were upregulated under Ni stress (Kim et al., 2005). GS and GOGAT activities were inhibited and the expression levels of their associated genes (*OsGS2, OsFd-GOGAT and OsNADH-GOGAT*) were downregulated in response to Ni stress.

It is known, that microRNAs in plants involved in the post-transcriptional regulation of genes expression and are critical regulators of HM stress (Dubey et al., 2018). So miR838 was found as the most responsive to the Ni- stress in *Ricinus communis* L (Celik & Akdaş, 2019). However, the role of microRNAs in Ni stress is poorly understood and new information to explore their role is necessary.

### Bacterial genes involved in Ni stress

Ni uptake by microorganisms is regulated by secondary transporters and by ATP-binding cassette (ABC) systems (Eitinger & Mandrand-Berthelot, 2000; Mulrooney & Hausinger, 2003; Maitra, 2016). The secondary systems - nickel/cobalt transporters (NiCoTs; TC 2.A.52.) are widely distributed in bacteria as well as in some archaea and fungi (Eitinger et al., 2005). The best investigated ABC-type Ni permease is NikABCDE system of *E. coli*, composed of a periplasmic binding protein (NikA), two integral membrane proteins (NikBC) and two ABC proteins (NikDE). In *E. coli*, Ni overstress is avoided *via* the repressor NikR, which binds to the promoter region of the *nik*ABCDE operon when Ni is present (De Pina et al., 1999; Chivers & Sauer, 2000). NikR has both strong (in the pM range) and weak (nM) Ni-binding sites, allowing to detect Ni at concentrations corresponding to the range from 1 to 100 molecules per cell (Bloom & Zamble, 2004; Maitra, 2016).

HupE/UreJ and UreH are two other families of suspected secondary metal carriers that are distantly related to NiCoTs (Eitinger et al., 2005). HupE/UreJ proteins are common among bacteria and encoded within certain hydrogenase (NiFe) or urease gene clusters (McMillan, Mau & Walker, 1998; Baginsky et al., 2004). Gene *ure*H was found in the urease operon in thermophilic bacteria (Maeda et al., 1994). These genes have similar sites to NiCoTs and presumably participate in Ni transport.

Often bacterial nickel resistance is plasmid mediated. For example, in resistant to heavy metals bacteria *Cupriavidus metallidurans* CH34 harbors plasmid pMOL28 which is responsible for Ni, Hg and Cr resistance (Nies et al., 1989; Mergeay, 1985). Ni efflux driven by a RND transporter is the basis of resistance in this strain. Two operon systems have been studied, a nickel-cobalt resistance Cnr (*cnr*CBA are structural resistance genes with *cnr*YXH regulatory genes) (Liesegang et al., 1993) and a Ni-Co-Cd resistance, Ncc (*nccCBA operon*) (Schmidt & Schlegel, 1989). The *atm*A gene (encodes ABC-transporter) was also found in the genome, which increases Ni and Co resistance in both *C. metallidurans* and *E. coli* and probably works together with other resistance operons (Mikolay & Nies, 2009).

Two distinct Ni resistance loci (*ncc* and *nre*) were found on plasmid pTOM9 from *Achromobacter xylosoxidans* 31A. Expression of the *nre*B gene was specifically induced by Ni and conferred Ni resistance on both *A. xylosoxidans* 31A and *E. coli* (Grass et al., 2001). Other resistant gene in *E.coli* is the *rcn*A (*yoh*M) gene responsible for Ni and Co efflux (Rodrigue, Effantin & Berthelot, 2005). In the unicellular cyanobacterium *Synechocystis* sp. PCC 6803 and *Helicobacter pylori*, a Ni resistance operon *nrs* and *czn* operon (Cd, Zn and Ni resistance) had been described respectively (García-Domínguez et al., 2000; Stahler et al., 2006). NrsB and NrsA proteins are homologues to CzcB and CzcA and they probably form a membrane-bound protein complex catalyzing Ni efflux by a proton/cation antiport.

Although bacterial genes involved in the transfer and accumulation of nickel have been studied, some questions remain unclear. For example, there is very little information on the genetic regulation in plant-bacterial associations. Plant-associated bacteria probably have a different genes enabling adaptation to the plant environment. The research in this direction is just emerging.

### Plant-bacterial associations

The rich diversity of root exudates and plant rhizodeposits attract diverse and unique microbial communities (Brencic & Winans, 2005; Chaparro et al., 2012). In plant-microbial associations, the host plant and associated microorganisms form a multicomponent integral system with new properties determined by the interaction of partners. Rhizobacteria can modulate their metabolism depending on the composition of root exudates towards optimizing nutrient acquisition (Hardoim, Van Overbeek & Van Elsas, 2008; Liu et al., 2019).

Root exudates and signal compounds that regulate the structure and diversity of the rhizosphere and rhizoplane microbial communities, and indirectly regulate the fluxes of biologically active substances synthesized by microorganisms (Bais et al., 2006; Smith, Gravel & Yergeau, 2017). Therefore plants can modulate its microflora by dynamically adapting it to the environment (Vandenkoornhuyse et al., 2015; Liu et al., 2019).

In its turn, rhizobacteria can modulate their metabolism depending on the composition of root exudates towards optimizing nutrient acquisition (Hardoim, Van Overbeek & Van Elsas, 2008). PGPR also can absorb ACC excreted from the plants and hydrolyzed by the ACC deaminase decreasing the content of ACC from the environment and consequently reduce stress ethylene level (Glick, 2005).

It was found, that *Pseudomonas putida, Pseudomonas fluorescens* can inhabit not only in soil, but on plant leaves and roots and form biofilms (Ude et al., 2006). The formation of the biofilm is influenced by the quorum-sensing (QS) process (Fuqua & Greenberg, 2002). The mechanisms of quorum formation are described in the review (Danhorn & Fuqua, 2007) AHLs (N-acyl-L-homoserine lactones) are the key components of QS signaling system (Danhorn & Fuqua, 2007; Ortíz-Castro et al., 2009). Plants identify AHLs and trigger changes in gene expression, defense responses of plants (Ma et al., 2016a).

Plants also can form in their roots specific symbiotic associations with microorganisms living in the spaces between cells of the root cortex and providing plants with nitrogen (such as plant-rhizobia and arbuscular mycorrhiza). The nitrogen-fixing rhizobia associated with legumes (Gray & Smith, 2005; Djordjevic, Mond-Radzman & Imin, 2015) as well as mycorrhizal fungi formed a symbiosis with the roots of most vascular plants are well understood (Upadhyaya et al., 2010; Emamverdian et al., 2015). The value of nitrogen fixation is very high for plants, and it is concluded that nitrogen fixation is of great ecological importance as a way to replenish the nitrogen available to plants in most natural ecosystems (Djordjevic, Mond-Radzman & Imin, 2015).

Bacteria, inhabiting in rhizosphere were classified according to their functional activities (Ahemed, 2019). The following groups PGPR were allocated: rhizomediators (solubilizing the HM and regulating HM availability), phytostimulators (stimulating plant growth because of phytohormone production), biofertilizers increasing soil nutrient availability, biopesticides (controlling plant pathogens and diseases) (Ahemed, 2019). All these properties are important in plant-microbial interactions under HM stress, including biocontrol function, which ensures the systemic resistance of plants (Ahemed, 2019; Manoj et al., 2020).

Despite a fairly long study of the microbial community of plants, our knowledge about it is quite limited (Quiza, St-Arnaud & Yergeau, 2015). Plant-associated bacteria probably have a different genes enabling adaptation to the plant environment. The studying in this direction is just beginning. Recently two sets of plant-associated bacteria genes (involved in plant colonization, and microbial competition between plant-associated bacteria) have been revealed in sequencing 484 bacterial genomes of bacterial isolates from roots of *Brassicaceae*, poplar, and maize (Levy et al., 2018). In addition to that 115 genes, which consist of 2% of all genes of *Pseudomonas simiae* (with colonization functions of the root system of *Arabidopsis thaliana*) were identified (Cole et al., 2017). A little earlier it has been shown, that wild accessions of *Arabidopsis thaliana* differ in their ability to form associations with *Pseudomonas fluorescens*, which effects on host health (Haney et al., 2015).

The new data, concerning plant-bacterial communication in the associations, such as plant-bacterial signaling in bacterial colonization of plant, quorum-sensing and biofilm formation, both in natural conditions and under Ni stress, increase our knowledge of plant-bacterial associations.

### Bacterial defense systems and PGPR mediated plant defense strategies

PGPR have developed some strategies to eliminate the inhibitory effects of HM toxicity (Qian et al., 2012; Babu, Kim & Oh, 2013; Ma et al., 2016a; Tiwari S. Lata, 2018). These strategies are speculated may be summarized schematically in (1) HM biosorption/precipitation by cell surface; (2) HM efflux pumping out of the cell by the transporter system; (3) HM binding in cell vacuole and other intracellular compartments; (4) exclusion of HM chelates into the extracellular space; and (5) enzymatic redox reaction *via* conversion of HM cations into a less toxic state. However, detoxification mechanisms are highly affected by the bacterial species and strains (Aktan, Tan & Icgen, 2013). Herewith one strain can simultaneously possess multiple defense mechanisms (Choudhury & Srivastava, 2001).

***Glutathione*,** intracellular polyphosphate granules, low and high molecular weight proteins and polyoxybutyric acid are involved in the defense system of bacteria when HM is absorbed by bacteria. However, the main defense mechanisms are realized outside bacterial cells, due to a change in the pH and redox potential of the medium, the mobilization of phosphates or the production of polysaccharides, siderophores and various antioxidant enzymes (Pishchik et al., 2016). The activity of glutathione-reductase was significantly increased in pea plants, (growing under Ni and Zn stresses) after the inoculation with *Rhizobium* sp. RP5 (Wani, Khan & Zaidi, 2008).

***Bacterial extracellular polysaccharides*** (EPS) can bind HM (Ahemad & Kibret, 2013), these substances can form complex with HM or by forming an effective barrier surrounding the cell (Rajkumar et al., 2010). Bacterial biofilms also may take part in sequestration or accumulation of Ni, and other HM, such as Al, Cd, Cu, Cr, Mn, Pb, Se, Zn (Khan et al., 2016). Endophytic bacterium *Caulobacter* sp. MN13 (alone and in combination with zeolite) reduced Ni uptake by sesame plants due to bacterial EPS and improved biochemical and agronomic parameters of plants (Naveed et al., 2020).

PGPR also produce a specific mixture of ***VOC***s (volatile organic compounds) that modulates plant growth hormones and plays important roles in their interactions with plants (Raza & Shen, 2020). It was shown that the rice inoculation with *Klebsiella variicola* F2, *Pseudomonas fluorescens* YX2 and *Raoultella planticola* YL2 lead to accumulation of GB (N,N,N-trimethyl glycine) and its precursor choline and improved water content in leaves (Gou et al., 2015). GB *in vivo* is both an effective osmoprotectant and a compatible solute (Felitsky et al., 2004). It was found also, that GB increased under Ni-stress (Sirhindi et al., 2016).

PGPR can secrete ***low molecular weight organic acids*** which increase Ni and other HM bioavailability for plant uptake (Becerra-Castro et al., 2011; Almaroai et al., 2012). A number of organic acids such as, citric, oxalic, malonic lactic etc. have chelating properties (Panhwar et al., 2013). The salts formed from these organic acids with heavy metals enter the plants. So, Ni-gluconate and Ni-citrate complexes were found to be present in the cocoa (Peeters et al., 2017). The potential of organic acids producing PGPR was highlighted in review (Rajkumar et al., 2012); however there is a controversial study, which did not show significant effect on the mobilization of HM (Evangelou, Ebel & Schaeffer, 2006; Park et al., 2011). This effect probably was attributed by increasing rhizosphere soil pH, or the presence of base cation saturations, which can decreased HM availability, as it was shown for Ni cations (Giovannetti et al., 2020).

***Biosurfactants*** are classified based on their biochemical nature or the producing microbial species. These natural compounds are classified into five major groups liposaccharides, lipopeptides, phospholipids, fatty acids (and neutral lipids), glycolipids (Sarubbo et al., 2015). PGPR strains from the genera *Acinetobacter*, *Pseudomonas* and *Bacillus* are found produced biosurfactants such as alasan, emulsan, glycolipid biosurfactant and surfactin (Sarubbo et al., 2015).

It was suggested, that biosurfactant molecules play a key role towards development and maintaining biofilms due to maintenance of water channels through the biofilm (Banat, De Rienzo & Quinn, 2014). Biosurfactants have been successfully employed in the remediation of environments contaminated with heavy metal ions (Sarubbo et al., 2015), *i.e.,* the lipopeptide biosurfactants from *Bacillus subtilis* A21 bound significant quantity of HM, including 32% Ni of polluted soil and was proposed for remediation (Singh & Cameotra, 2013).

***Siderophores.*** Because Fe (II) is highly toxic in its free form due to its participation in the Fenton reaction, and Fe (III) is insoluble in solutions and not bioavailable (Miethke & Marahiel, 2007), the microorganisms have developed an iron absorption strategy through siderophores (Rajkumar et al., 2012; Ashraf et al., 2017; Deicke et al., 2019; Hofmann, Morales & Tischler, 2020). Siderophore is also reported to suppress the plant pathogens in different plants, such as tomato (Aznar & Dellagi, 2015), pepper (Yu et al., 2011) and maize (Pal et al., 2001) due to its participation in plants induce systemic resistance (ISR) (Bakker, Pieterse & Van Loon, 2007; Ghosh, Bera & Chakrabarty, 2020). Siderophores are low molecular - weight metabolites (500–1,500 daltons) with high affinities for Fe^3+^ with stability constants (Andrews, RobinsonA & Rodríguez-Quiñones, 2003). Depending on the functional group siderophores are generally classified in different groups: hydroxamates, catecholates (including phenolates), carboxylates and mixed type siderophores (Hofmann, Morales & Tischler, 2020). However, their structural nature is variable and they bind different metals and even metalloids (Retamal-Morales et al., 2018). Such complexes are transported into the periplasm by TonB-dependent transporters (TBDT), and are transported across the plasma membrane by ATP-binding cassette (ABC) transporters in both Gram-negative and Gram-positive bacteria (Ghssein & Matar, 2018). Other metallophores are found and described also for their ability to uptake metals other than iron, such as, for example, nickelophore for nickel (Lebrette et al., 2015), and zincophore for zinc (Bobrov et al., 2017). However, the complexes with Ni are stable, compared to complexes with Fe (Hofmann, Morales & Tischler, 2020). Bacteria can produce more than one siderophore, so *Pseudomonas aeruginosa* produces pyoverdine and pyochelin (Minandri et al., 2016). The new metallophor pseudopaline from *Pseudomonas aeruginosa* is known more specific for the chelation of nickel and zinc (Lhospice et al., 2017).

In addition to the main function of supplying microorganisms and plants with iron, other functions of siderophores are described in the literature. We will focus on the most interesting ones concerning the bacterial protective properties against HM stress. The signaling function of siderophores is discussed in literature (Roux, Payne & Gilmore, 2009; Johnstone & Nolan, 2015). It is suggested, that the siderophore itself, or a metal complex thereof, acts directly as a signaling molecule or a mediator of quorum sensing (Roux, Payne & Gilmore, 2009; Dembitsky, Quntar & Srebnik, 2011).

PGPR, producing siderophores, generally increase HM bioavailability through complexation reactions (Khan et al., 2017; Sujkowska-Rybkowska et al., 2020). Therefore such PGPR can be used in phytoremediation to improve the phytoextraction of Ni and other HM. It was found that more than 80% of endophytic bacteria increased the production of siderophores in the presence of heavy metals (Ni and Co, Cr, Cu, Zn) and also reduced metal toxicity in their host plant *Alyssum bertolonii* (Ma et al., 2011a). Ni- resistant *Pseudomonas sp*. A3R3 increased plants biomass as well as Ni accumulation in *Brassica juncea* and *A. serpyllifolium*, due to ACC-deaminase, siderophores, IAA activities (Ma et al., 2011b).

However, there are the opposite evidences too, that bacterial siderophores bound Ni cations (so as Pb and Zn), decreased Ni contents in plants and protecting plants against HM toxicity (Burd, Dixon & Glick, 1998; Dimkpa et al., 2008; Dimkpa, Weinand & Asch, 2009; Tank & Saraf, 2009), or did not influence on the HM concentrations in plants (Kuffner et al., 2010).

The literature analysis allows us to assume that the mechanisms, determining HM uptake by plants with the participation of bacterial siderophores are still remaining unknown. Moreover, there is a need to isolate and analyze new siderophores from different PGPR for the application of these PGPR both in bioremediation and plant protection against biotic stresses.

***Bacterial phytohormones*** The PGPR regulate the nutritional and hormonal balance in plants and induce plant tolerance to stress (Spence & Bais, 2015). The phytohormones synthesis in plant rhizosphere is a mechanism of improvement of plant growth under stress (Etesami, Alikhani & Hosseini, 2015).

The participation of microbial auxins in changing of plant root morphology is well studied. Microbial phytohormones affect the metabolism of endogenous growth regulators in plant tissue and change root morphology under heavy metal stress conditions. Auxin-producing PGPR reduced the effect of HM stress on physiological processes in plants (Pishchik et al., 2009). Auxin- producing *B. megaterium* MCR-8 increased growth, contents of total phenols, flavonoids, and activities of SOD, CAT, POD, and APX in inoculated *Vinca rosea* plants under Ni stress (Khan et al., 2017). Although we found very little literature strongly supporting the involvement of PGPR hormones in plant Ni stress management, we can speculate this topic, based on the non-specifically plant reactions on the abiotic stress. Overcoming some of the adverse effects of Ni-stress can help plants cope with stress in general. For, example, abscisic acid (ABA) is plant hormone regulated of water misbalance in plants by controlling stomatal closure and stress signal transduction pathways (Kudoyarova, Kholodova & Veselov, 2013). Therefore we may suggest that ABA-producing PGPR can help plants overcome water misbalance caused by Ni stress.

Cytokinin-producing bacteria *Bacillus subtilis* IB-22 increased auxin production by wheat roots as well as stimulate root exudation of amino acids. Authors proposed that the ability of rhizobacteria to produce cytokinins and thereby stimulate amino acids rhizodeposition may be important in enhancing rhizobacterial colonization of the rhizoplane (Kudoyarova et al., 2014). It was shown that cytokinin-producing *B. subtilis* increased root biomass and cytokinin concentration in leaves of *Platycladus orientalis* by 47.5% under water stress. Cytokinin in plant tissue promoted stomatal opening and mitigated some of the harmful effects of water stress (Liu et al., 2013). It also have been reported that cytokinin-producing bacteria from genera *Arthrobacter, Azospirillum, Bacillus* and *Pseudomonas* increased proline content in plant tissue of soybean and shoot and root biomass under salt stress (Naz, Bano & Ul-Hassan, 2009). The applying of cytokinin-producing PGPR may be useful in plant protection under Ni-stress. *Bacillus megaterium* LZR216 produced cytokinins, changed root morphogenesis of *Arabidopsis thaliana* and regulated the transcriptional level of cytokinin-responsive environmental sensor AHK3/AHK4 in *Arabidopsis thaliana*. The intact cytokinin-signaling pathway is necessary for PGPR -promoted plant growth and root system architecture alteration (Jianfeng et al., 2017).

**Figure 1:**
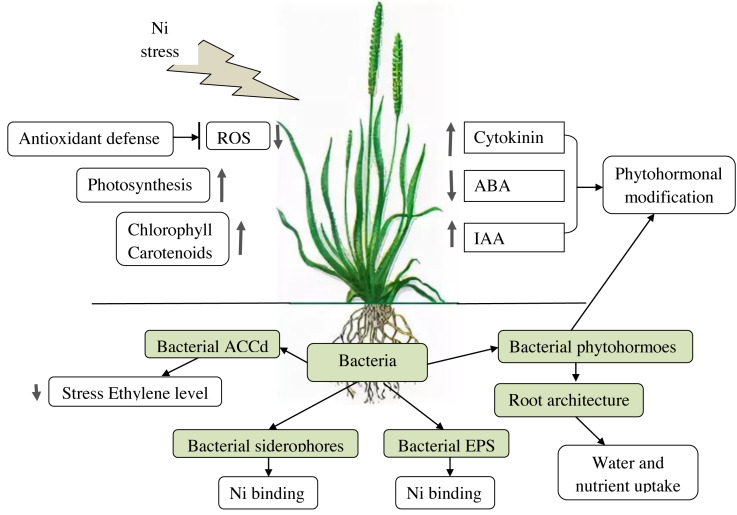
Effects of PGPB on plants under Ni-stress.

PGPB effects on plants under Ni stress are summarized in Table 2 and in Fig. 1.

**Table 2:** Effect of PGPB on plants under Ni stress.

**Plant species**	**Bacteria**	**Effect**	**References**
*Brassica campestris* L.	*Kluyvera ascorbata* SUD165	Bacterium displayed ACC deaminase activity, and produced siderophores, and decreased the level of stress ethylene induced by the Ni	Burd, Dixon & Glick (1998)
*Brassica juncea* L.	*Bacillus subtilis* SJ-101	Bacterium produced IAA and stimulated of Ni-phytoextraction	Zaidi et al. (2006)
*Brassica juncea* L.	*Psychrobacter* sp. SRA1, *Bacillus cereus* SRA10	Bacteria displayed ACC deaminase activity, produced a siderophores and IAA, increased the accumulation of Ni in the root and shoot tissues	Ma, Rajkumar & Freitas (2009)
*Brassica juncea* L.; *Brassica oxyrrhina* Coss	*Psychrobacter* sp. SRA2	Bacterium displayed ACC deaminase activity, produced a siderophores and IAA, increased the fresh and dry biomass of the plants	
*Brassica juncea* L.; *Ricinus communis* L.	*Pseudomonas* sp. A3R3, *Psychrobacter* sp. SRS8	Bacteria improved plant biomass production and decreased heavy metal accumulation	Ma et al. (2015)
*Brassica juncea* L.; *Luffa cylindrical* L.; *Sorghum halepense* L.	*Bacillus megaterium* SR28C	Bacterium alleviated the detrimental effects of Ni by reducing its uptake and translocation to the plants	Rajkumar, Ma & Freitas (2013)
*Vigna unguiculata* L.	*Streptomyces acidiscabies* E13	Bacterium produced hydroxamate siderophores and promoted plant growth under Fe- and Ni- stress	Dimkpa et al. (2008)
*Vinca rosea* L.	*Bacillus megaterium* MCR-8	Bacterium alleviated Ni stress, increased the accumulation of total phenols, flavonoids and enzymes SOD, CAT, POD, APX, improved phytoextraction	Khan et al. (2017)
*Oryza sativa* L.	*Enterobacter ludwigii* SAK5, * Exiguobacterium indicum* SA22	Bacteria increased plant growth parameters under Cd and Ni stress, also enhance glutathione, proline, and sugar content	Jan et al. (2019)
*Cicer arietinum* L.	*Pseudomonas aeruginosa* SFP1	Bacterium declined the level of stress markers (proline and APX, SOD, CAT, and GR), as well as with Cr (VI) and Ni uptake by plants	Saif & Khan (2018)
*Rafanus sativus* L.	*Bacillus* sp. CIK-516	Bacterium produced IAA, and displayed ACC deaminase activity, and increased Ni uptake by radish	Akhtar et al. (2018)
*Triticum aestivum*	*Bacillus subtilis BM2*	Bacterium displayed ACC deaminase activity, produced IAA, siderophores, ammonia. Bacterium increased plant growth parameters under Ni stress, decreased Ni content in plants and decrease SOD, GR and CAT activity	Rizvi et al. (2019)

**Notes.**

ACC deaminase 1-aminocyclopropane-1-carboxylic acid deaminase IAA indole-3-acetic acid SOD superoxide dismutase CAT catalase POD peroxidase activity APX ascorbate peroxidase GR glutathione reductase

### Bacterial effects on plant stress-responsive genes

Bacteria regulate major metal responsible and transporter genes expression (Manoj et al., 2020). It was revealed, that the inoculation with endophytic bacteria *Enterobacter ludwigii* SAK5 and *Exiguobacterium indicum* SA22 led to increasing of Ni content in rice plants, however the expression level of stress-responsive genes, such as *OsGST* (glutathione-s transferase), *OsMTP1* (HM transporting), and *OsPCS1* (phytochelatin synthases) level was lower in treated inoculated plants than in treated non-inoculated plants which indicated a decrease in stress levels when inoculated with bacteria (Jan et al., 2019). However, the studies of bacterial effect on gene expression in plants under Ni stress are insufficient and further studies are needed. The missing information on micro RNAs mediated by bacterial inoculation under nickel stress is also necessary.

### Bioremediation

Taking into consideration the differences in responses of plants and microorganisms to HM, bioremediation employs two different approaches: phytoextraction and phytostabilization. Phytoextraction implies the use of HM-accumulating plants that can accumulate metals in aboveground organs, which are then utilized. Phytostabilization (the conversion of chemicals to less mobile and active forms) can employ plants with high resistance to HM, localizing HM mainly in the root system (Chaney & Mahoney, 2014). Phytoremediation, an emerging technique makes use of plants and their associated microbes to clean up heavy metals pollutant from soil (Kumar & Verma, 2018). In addition phytoremediation is cost effective and is a more sustainable approach for removal of HM (Ma et al., 2016b).

*Streptanthus polygaloides* A. Gray, a Ni- hyperaccumulating plant from *Brassicaceae* family was successfully used in phytoextraction. The shoot Ni concentration of *S. polygaloides* averaged 5,300 mg kg^−^^1^, whereas Ni concentration in soil was of 3,340 mg kg^−^^1^ (Nicks & Chambers, 1995).

During development of Ni phytoextraction technology mean Ni concentrations in the shoots *Alissum murale* and *Alissum corsicum* ranged from 4,200 to 20,400 mg kg^−^^1^. Ni uptake by these Alyssum species was reduced in the field experiments at lower soil pH and increased at higher soil pH, and that was an uninspected result (Li et al., 2003). Nowadays eight species of *Alissum* (family *Brassicaceae)* described as Ni-hyperaccumulators (Pollard, Reeves & Baker, 2014).

## Conclusions

The review highlights the importance of bacterial contribution in plant protection under Ni-stress. The understanding of the mechanisms of bacterial plant defense against nickel stress in plant-bacterial associations has been formed in recent decades. Bacteria activated numerous genes in plants in response to Ni- stress; however research in this direction is just emerging. Moreover, intensive study of plant genes involved in protection against nickel stress has also taken place in the last decades, when some plant genes have been identified and proposed as candidates for plant protection against nickel stress. Despite the fact that various mechanisms of bacterial protection have already been described in the literature, some issues remain unexplored. So, more detailed studies of the effect of bacterial phytohormones on plants under Ni stress is required for understanding. More information concerning plant-microbial crosstalk in response to Ni-stress is missing; therefore omics-based technologies, such as transcriptomics, proteomics and metabolomics must be used in future experiments to decipher the mechanisms of bacterial protection of plants. Further study of environmental conditions is also necessary, since the effectiveness of the protective actions of bacteria is also determined by soil conditions and the magnitude of the stress load. The review of the scientific data results on the ability of plant-microbial associations to regulate the nickel uptake by plants with subsequent utilization of plant biomass will help to develop bioremediation technologies for polluted lands, or to produce eco-friendly agricultural crops on HM contaminated soils (with the level of Ni and other HM contents not exceeding the maximum permissible concentrations).

## Additional Information and Declarations

### Data Availability

The following information was supplied regarding data availability: There are no raw data or code because this article is a Literature Review.

## References

[ref-1] Ahemad M, Kibret M (2013). Recent trends in microbial biosorption of heavy metals: a review. Biochemistry & Molecular Biology.

[ref-2] Ahemed M (2019). Remediation of metalliferous soils through the heavy metal resistant plant growth promoting bacteria: paradigms and prospects. Arabian Journal of Chemistry.

[ref-3] Aina R, Labra M, Fumagalli P, Vannini C, Marsoni M, Cucchi U, Bracale M, Sgorbati S, Citterio S (2007). Thiol-peptide level and proteomic changes in response to cadmium toxicity in *Oryza sativa* L. roots. Environmental and Experimental Botany.

[ref-4] Aina R, Sgorbati S, Santagostino A, Labra M, Ghiani A, Citterioet S (2004). Specific hypomethylation of DNA is induced by heavy metals in white clover and industrial hemp. Physiologia Plantarum.

[ref-5] Akhtar MJ, Ullah S, Ahmad I, Rauf A, Nadeem SM, Khan MY, Hussain S, Bulgariue L (2018). Nickel phytoextraction through bacterial inoculation in *Raphanus sativus*. Chemosphere.

[ref-6] Aktan Y, Tan S, Icgen B (2013). Characterization of lead-resistant river isolate *Enterococcus Faecalis* and assessment of its multiple metal and antibiotic resistance. Environmental Monitoring and Assessment.

[ref-7] Almaroai Y, Usman AR, Ahmad M, Kim KR, Moon DH, Lee SS, Ok YS (2012). Effects of synthetic chelators and low-molecular-weight organic acids on chromium, copper, and arsenic uptake and translocation in maize (*Zea mays* L.). Soil Science.

[ref-8] Ameen N, Amjad M, Murtaza B, Abbas Gh, Shahid M, Imran M, Naeem MA, Niazi NK (2019). Biogeochemical behavior of nickel under different abiotic stresses: toxicity and detoxification mechanisms in plants. Environmental Science and Pollution Research.

[ref-9] Amjad M (2020). Nickel toxicity induced changes in nutrient dynamics and antioxidant profiling in two maize (*Zea mays* L.) hybrids. Plants.

[ref-10] Amjad M, Ameen N, Murtaza B, Imran M, Shahid M, Abbas G, Naeem MA, Jacobsen S-E (2020). Comparative physiological and biochemical evaluation of salt and nickel tolerance mechanisms in two contrasting tomato genotypes. Physiologia Plantarum.

[ref-11] Andrews SC, RobinsonA K, Rodríguez-Quiñones F (2003). Bacterial iron homeostasis. FEMS Microbiology Reviews.

[ref-12] Arefifard M, Mahdieh M, Amirjani M (2014). Study of the effect of nickel heavy metals on some physiological parameters of *Catharanthus roseus*. Natural Product Research.

[ref-13] Ashraf MA, Hussain I, Rasheed R, Iqbal M, Riaz M, Arif MS (2017). Advances in microbe-assisted reclamation of heavy metal contaminated soils over the last decade: a review. Journal of Environmental Management.

[ref-14] Ashraf MY, Sadiq R, Hussain M, Ashraf M, Ahmad MSA (2011). Toxic effect of nickel (Ni) on growth and metabolism in germinating seeds of sunflower (*Helianthus annuus* L.). Biological Trace Element Research.

[ref-15] Aziz E, Gad N, Badran N (2007). Effect of cobalt and Ni on plant growth, yield and flavonoids content of *Hibiscus sabdariffa* L. Australian Journal of Basic and Applied Sciences.

[ref-16] Aznar A, Dellagi A (2015). New insights into the role of siderophores as triggers of plantimmunity: what can we learn from animals?. Journal of Experimental Botany.

[ref-17] Babu AG, Kim JD, Oh BT (2013). Enhancement of heavy metal phytoremediation by *Alnus firma* with endophytic *Bacillus thuringiensis* GDB-1. Journal of Hazardous Materials.

[ref-18] Baginsky C, Palacios JM, Imperial J, Ruiz-Argueso T, Brito B (2004). Molecular and functional characterization of the *Azorhizobium caulinodans* ORS571 hydrogenase gene cluster. FEMS Microbiology Letters.

[ref-19] Bais HP, Weir TL, Perry LG, Gilroy S, Vivanco JM (2006). The role of root exudates in rhizosphere interactions with plants and other organisms. Annual Review of Plant Biology.

[ref-20] Bakker PAHM, Pieterse CM, Van Loon LC (2007). Induced systemic resistance by fluorescent *Pseudomonas* spp. Phytopathology.

[ref-21] Balal R, Shahid M, Javaid M, Anjum M, Ali H, Mattson N, Garcia-Sanchez F (2016). Foliar treatment with *Lolium perenne* (Poaceae) leaf extract alleviates salinity and nickel-induced growth inhibition in pea. Brazilian Journal of Botany.

[ref-22] Banat IM, De Rienzo MAD, Quinn GA (2014). Microbial biofilms: biosurfactants as antibiofilm agents. Applied Microbiology and Biotechnology.

[ref-23] Becerra-Castro C, A. Prieto-Fernández A, V. Álvarez Lopez V, Monterroso C, Cabello-Conejo MI, Acea MJ, Kidd PS (2011). Nickel solubilizing capacity and characterization of rhizobacteria isolated from hyperaccumulating and non-hyperaccumulating subspecies of *Alyssum serpyllifolium*. International Journal of Phytoremediation.

[ref-24] Bhatia NP, Walsh KB, Baker AJM (2005). Detection and quantification of ligands involved in nickel detoxification in a herbaceous Ni hyperaccumulator *Stackhousia tryonii Bailey*. Journal of Experimental Botany.

[ref-25] Bloom SL, Zamble DB (2004). Metal-Selective DNA-Binding Response of Escherichia coli NikR. Biochemistry.

[ref-26] Bobrov AG, Kirillina O, Fosso MY, Fetherston JD, Miller MC, Van Cleave TT, Burlison JA, ArnoldW K, Lawrenz MB, Garneau-Tsodikova S, Perry RD (2017). Zinc transporters YbtX and ZnuABC are required for the virulence of *Yersinia pestis* in bubonic and pneumonic plague in mice. Metallomics.

[ref-27] Brencic A, Winans SC (2005). Detection and response to signals involved in host-microbe interactions by plant-associated bacteria. Microbiology and Molecular Biology Review.

[ref-28] Burd GI, Dixon DG, Glick BR (1998). Plant growth-promoting bacterium that decreases nickel toxicity in seedlings. Applied and Environmental Microbiology.

[ref-29] Cabanillas A, Ginebreda D, Guillén E, Martínez Barceló D, Moragas L, Robusté J, Darbra RM (2012). Fuzzy logic based risk assessment of effluents from waste-water treatment plants. Science of the Total Environment.

[ref-30] Cabello-Conejo MI, Prieto-Fernández Á, Kidd PS (2014). Exogenous treatments with phytohormones can improve growth and nickel yield of hyperaccumulating plants. Science of the Total Environment.

[ref-31] Cabello-Conejo MI, Centofanti T, Kidd PS, Prieto-Fernández Á, Chaney R (2013). Evaluation of plant growth regulators to increase nickel phytoextraction by *Alyssum* species. International Journal of Phytoremediation.

[ref-32] Callahan DL, Roessner U, Dumontet V, De Livera AM, Doronil A, Baker AJM, Kolev SD (2012). Elemental and metabolite profiling of nickel hyperaccumulators from New Caledonia. Phytochemistry.

[ref-33] Celik Ö, Akdaş EY (2019). Tissue-specific transcriptional regulation of seven heavy metal stress-responsive miRNAs and their putative targets in nickel indicator castor bean (*R. communis* L.) plants. Ecotoxicology and Environmental Safety.

[ref-34] Centofanti T, Siebecker M, Chaney R, Davis A, Sparks D (2012). Hyperaccumulation of nickel by *Alyssum corsicum* is related to solubility of Ni mineral species. Plant and Soil.

[ref-35] Chaney RL, Mahoney M (2014). Phytostabilization and phytomining: principles and successes. Meeting proceedings. Proc. life of mines conference, July 15-17.

[ref-36] Chaparro JM, Sheflin AM, Manter DK, Vivanco JM (2012). Manipulating the soil microbiome to increase soil health and plant fertility. Biology and Fertility of Soils.

[ref-37] Chen L, Guo Y, Yang L, Wang Q (2008). Synergistic defensive mechanism of phytochelatins and antioxidative enzymes in *Brassica chinensis* L. against Cd stress. Chinese Science Bulletin.

[ref-38] Chivers PT, Sauer RT (2000). Regulation of high affinity nickel uptake in bacteria.Ni -dependent interaction of NikR with wild-type and mutant operator sites. Journal of Biological Chemistry.

[ref-39] Choudhury R, Srivastava S (2001). Zinc resistance mechanisms in bacteria. Current Science.

[ref-40] Cobbett CS (2000). Phytochelatins and their roles in heavy metal detoxification. Plant Physiology.

[ref-41] Cole BJ, Feltcher ME, Waters RJ, Wetmore KM, Mucyn TS, Ryan EM, Gaoyan Wang G, Ul-Hasan S, McDonald M, Yoshikuni Y, Rex R, Malmstrom RR, Deutschbauer AM, Dangl JL, Visel A (2017). Genome-wide identification of bacterial plant colonization genes. PLOS Biology.

[ref-42] Czajka K, Michael P, Nkongolo KK (2018). High level of nicotianamine synthase (NAS3) and natural resistance associated macrophage protein (NRAMP4) gene transcription induced by potassium nitrate in trembling aspen (*Populus tremuloides*). American Journal of Biochemistry and Biotechnology.

[ref-43] Dalvi AA, Bhalerao SA (2013). Response of plants towards heavy metal toxicity: an overview of avoidance, tolerance and uptake mechanism. Annals of Plant Sciences.

[ref-44] Danhorn T, Fuqua C (2007). Biofilm formation by plant-associated bacteria. Annual Review of Microbiology.

[ref-45] Deicke M, Mohr JF, Roy S, Herzsprung P, Bellengerd JP, Wichard T (2019). Metallophore profiling of nitrogen-fixing *Frankia* spp. to understand metal management in the rhizosphere of actinorhizal plants. Metallomics.

[ref-46] Dembitsky VM, Al Quntar AAA, Srebnik M (2011). Natural and synthetic small boron-containing molecules as potential inhibitor of bacterial and fungal quorum sensing. Chemical Reviews.

[ref-47] Deng THB, Van der Ent A, Tang YT, Sterckeman T, Echevarria G, Morel J-L, Qiu R-L (2018). Nickel hyperaccumulation mechanisms: a review on the current state of knowledge. Plant and Soil.

[ref-48] De Pina K, Desjardin V, Mandrand-Berthelot M-A, Giordano G, Wu L-F (1999). Isolation and characterization of the *nikR* gene encoding a nickel-responsive regulator in *Escherichia coli*. Journal of Bacteriology.

[ref-49] Dimkpa C, Svatoš A, Merten D, Büchel G, Kothe E (2008). Hydroxamate siderophores produced by *Streptomyces acidiscabies* E13 bind nickel and promote growth in cowpea (*Vigna unguiculata* L.) under nickel stress. Canadian Journal of Microbiology.

[ref-50] Dimkpa C, Weinand T, Asch F (2009). Plant–rhizobacteria interactions alleviate abiotic stress conditions plant. Cell and Environment.

[ref-51] Ding H, Nan Z, Liu X, Li Y, Wang S, Qin S, Zhao Z-J (2008). Characteristics of selected heavy metal pollution in suburb cropland, Jinchang City, Gansu, China. Journal of Agro-Environment Science.

[ref-52] Djeukam CL, Nkongolo K (2018). Expression of genes associated with nickel resistance in red oak (*Quercus rubra*) populations from a metal contaminated region. Bulletin of Environmental Contamination and Toxicology.

[ref-53] Djordjevic MA, Mond-Radzman NA, Imin N (2015). Small-peptide signals that control root nodule number, development, and symbiosis. Journal of Experimental Botany.

[ref-54] Dubey S, Shri M, Gupta A, Rani V, Chakrabarty D (2018). Toxicity and detoxification of heavy metals during plant growth and metabolism. Environmental Chemistry Letters.

[ref-55] Egamberdieva D, Abd-Allah EF, Da Silva JTA, Parvaiz A (2016). Microbially assisted phytoremediation of heavy metal–contaminated soils. Plant metal interaction. Emerging remediation techniques.

[ref-56] Eitinger T, Mandrand-Berthelot MA (2000). Nickel transport systems in microorganisms. Archives of Microbiology.

[ref-57] Eitinger T, Suhr J, Moore L, Smith JA (2005). Secondary transporters for nickel and cobalt ions: theme and variations. BioMetals.

[ref-58] Emamverdian A, Ding Y, Mokhberdoran F, Xie Y (2015). Heavy metal stress and some mechanisms of plant defense response. Scientific World Journal.

[ref-59] Etesami H, Alikhani HA, Hosseini HM (2015). Indole-3-acetic acid (IAA) production trait, a useful screening to select endophytic and rhizosphere competent bacteria for rice growth promoting agents. Methods X.

[ref-60] Evangelou MWH, Ebel M, Schaeffer A (2006). Evaluation of the effect of small organic acidson phytoextraction of Cu and Pb from soil with tobacco *Nicotiana tabacum*. Chemosphere.

[ref-61] Evdokimova GA, Kalabin GV, Mozgova NP (2011). Contents and toxicity of heavy metals in soils of the zone affected by aerial emissions from the severonikel enterprise. Eurasian Soil Science.

[ref-62] Fabiano CC, Tezotto T, Favarin JL, Polacco JC, Mazzafera P (2015). Essentiality of nickel in plants: a role in plant stresses. Frontiers in Plant Science.

[ref-63] Felitsky DJ, Cannon JG, Capp MW, Hong J, Van Wynsberghe AW, Anderson CF, Record MT (2004). The exclusion of glycine betaine from anionic biopolymer surface: why glycine betaine is an effective osmoprotectant but also a compatible solute. Biochemistry.

[ref-64] Ferraz P, Fidalgo F, Almeida A, Teixeira J (2012). Phytostabilization of nickel by the zinc and cadmium hyperaccumulator *Solanum nigrum* L. are metallothioneins involved?. Plant Physiology and Biochemistry.

[ref-65] Fourati E, Wali M, Vogel-Mikuš K, Abdelly C, Ghnaya T (2016). Nickel tolerance, accumulation and subcellular distribution in the halophytes *Sesuvium portulacastrum* and *Cakile maritima*. Plant Physiology and Biochemistry.

[ref-66] Freeman JL, Garcia D, Kim D, Hopf A, Salt DE (2005). Constitutively elevated salicylic acid signals glutathione-mediated nickel tolerance in *Thlaspi* nickel hyperaccumulators. Plant Physiology.

[ref-67] Freeman J, Persans M, Nieman K, Albrecht C, Peer W, Pickering IJ, Salt D (2004). Increased glutathione biosynthesis plays a role in nickel tolerance in *Thlaspi* nickel hyperaccumulators. The Plant Cell.

[ref-68] Fuqua C, Greenberg EP (2002). Listening in on bacteria: acyl-homoserine lactone signalling. Nature Reviews Molecular Cell Biology.

[ref-69] Gajewska E, Skłodowska M (2005). Antioxidative responses and proline level in leaves and roots of pea plants subjected to nickel stress. Acta Physiologiae Plantarum.

[ref-70] Gajewska E, Skłodowska M (2007). Effect of nickel on ROS content and antioxidative enzyme activities in wheat leaves. Biometals.

[ref-71] Gajewska E, Skłodowska M (2009). Nickel-induced changes in nitrogen metabolism in wheat shoots. Journal of Plant Physiology.

[ref-72] Gajewska E, Skłodowska M, Słaba M, Mazur J (2006). Effect of nickel on antioxidative enzyme activities, proline and chlorophyll contents in wheat shoots. Biologia Plantarum.

[ref-73] García-Domínguez M, Lopez-Maury L, Florencio FJ, Reyes JC (2000). A gene cluster involved in metal homeostasis in the cyanobacterium *Synechocystis* sp. strain PCC 6803. Journal of Bacteriology.

[ref-74] Gerendas J, Zhu Z, Sattelmacher B (1998). Influence of N and Ni supply on nitrogen metabolism and urease activity in rice *Oryza sativa* L. Journal of Experimental Botany.

[ref-75] Ghasemi R, Ghaderian SM, Krämer U (2009). Interference of nickel with copper and iron homeostasis contributes to metal toxicity symptoms in the nickel hyperaccumulator plant *Alyssum inflatum*. New Phytologist.

[ref-76] Ghosh SK, Bera T, Chakrabarty AM (2020). Microbial siderophore –A boon to agricultural sciences. Biological Control.

[ref-77] Ghssein G, Matar SF (2018). Chelating mechanisms of transition metals by bacterial metallophores pseudopaline and staphylopine: a quantum chemical assessment. Computation.

[ref-78] Ghosh M, Singh SP (2005). A review phytoremediation of heavy metals and utilization of it’s by-products. Applied Ecology and Environmental Research.

[ref-79] Giovannetti F, Elcio MM, Santos F, Lavresa A (2020). Agricultural crop influences availability of nickel in the rhizosphere; a study on base cation saturations, Ni dosages and crop succession. Rhizosphere.

[ref-80] Glick BR (2005). Modulation of plant ethylene levels by the bacterial enzyme ACC deaminase. FEMS Microbiology Letters.

[ref-81] Gou W, Tian L, Ruan Z, Zheng P, Chen F, Zhang L, Cui Z, Zheng P, Li Z, Gao M, Shi W, Zhang L, Liu J, Hu J (2015). Accumulation of choline and glycinebetaine and drought stress tolerance induced in maize (*Zea mays*) by three plant growth promoting rhizobacteria (PGPR) strains. Pakistan Journal of Botany.

[ref-82] Grass G, Fan B, Rosen BP, Lemke K, Schlegel HG, Rensing C (2001). NreB from *Achromobacter xylosoxidans* 31A is a nickel-induced transporter conferring nickel resistance. Journal of Bacteriology.

[ref-83] Gray EJ, Smith DL (2005). Intracellular and extracellular PGPR: commonalities and distinctions in the plant–bacterium signaling processes. Soil Biology & Biochemistry.

[ref-84] Guo L, Ding Y, Xu Y, Li Z, Jin Y, He K, Fang Y, Zhao H (2017). Responses of *Landoltia punctata* to cobalt and nickel: removal, growth, photosynthesis, antioxidant system and starch metabolism. Aquatic Toxicology.

[ref-85] Guo J, Xu L, Su Y, Wang H, Gao S, Xu J, Que Y (2013). ScMT2-1-3, a metallothionein gene of sugarcane, plays an important role in the regulation of heavy metal tolerance/accumulation. BioMed Research International.

[ref-86] Gupta V, Jatav PK, Verma R, Kothari S, Kachhwaha S (2017). Nickel accumulation and its effect on growth, physiological and biochemical parameters in millets and oats. Environmental Scienceand Pollution Research.

[ref-87] Hall JL (2002). Cellular mechanisms for heavy metal detoxification and tolerance. Journal of Experimental Botany.

[ref-88] Haney CH, Samuel BS, Bush J, Ausubel FM (2015). Associations with rhizosphere bacteria can confer an adaptive advantage to plants. Nature Plants.

[ref-89] Haq NU, Raza S, Luthe DS, Heckathorn SA, Shakeel SN (2013). A dual role for the chloroplast small heat shock protein of *Chenopodium album* including protection from both heat and metal stress. Plant Molecular Biology Reporter.

[ref-90] Hardoim PR, Van Overbeek LS, Van Elsas JD (2008). Properties of bacterial endophytes and their proposed role in plant growth. Trends in Microbiology.

[ref-91] Hauser M-T (2014). Molecular basis of natural variation and environmental control of trichome patterning. Frontiers in Plant Science.

[ref-92] Helaoui S, Boughattas I, Hattab S, Mkhinini M, Alphonse V, Livet A, Bousserrhine N, Banni M (2020). Physiological, biochemical and transcriptomic responses of *Medicago sativa* to nickel exposure. Chemosphere.

[ref-93] Hofmann M, Morales RG, Tischler D (2020). Metal binding ability of microbial natural metal chelators and potential applications. Natural Product Reports.

[ref-94] Hussain MB, Ali S, Azam A, Hina S, Ahsan M, Farooq BA, Bharwana SA, Gill MB (2013). Morphological, physiological and biochemical responses of plants to nickel stress: a review. African Journal of Agricultural Research.

[ref-95] Idrees M, Naeema M, Aftab T, Khan MMA (2013). Salicylic acid restrains nickel toxicity, improves antioxidant defense system and enhances the production of anticancer alkaloids in *Catharanthus roseus* (L.). Journal of Hazardous Material.

[ref-96] Idris R, Trivonova R, Puschenreiter M, Wenzel WW, Sessitsch A (2004). Bacterial communities associated with flowering plants of the Ni-hyperaccumulator *Thlaspi goesingense*. Applied and Environmental Microbiology.

[ref-97] Ihedioha JN, Ukoha PO, Ekere NR (2017). Ecological and human health risk assessment of heavy metal contamination in soil of a municipal solid waste dump in Uyo, Nigeria. Environmental Geochemistry and Health.

[ref-98] Jan R, Khan MA, Asaf S, Lubna, Lee I-J, Kim KM (2019). Metal resistant endophytic bacteria reduces cadmium, nickel toxicity, and enhances expression of metal stress related genes with improved growth of *Oryza sativa*, via regulating its antioxidant machinery and endogenous hormones. Plants.

[ref-99] Jaskulak M, Rorat A, Grobelak A, Chaabene Z, Małgorzata Kacprzak M, Vandenbulcke F (2019). Bioaccumulation, antioxidative response, and metallothionein expression in *Lupinus luteus* L. exposed to heavy metals and silver nanoparticles. Environmental Science and Pollution Research.

[ref-100] Jianfeng W, Zhang Y, Jin J, Li Q, Chenzhou Z, Wenbin N, Xiaomin W, Rongrong M, Yurong B (2017). An intact cytokinin-signaling pathway is required for Bacillus sp. LZR216-promoted plant growth and root system architecture alteration in Arabidopsis thaliana seedlings. Plant Growth Regulation.

[ref-101] Jing Y, He Z, Yang X (2007). Role of soil rhizobacteria in phytoremediation of heavy metal contaminated soils. Journal of Zhejiang University Science B.

[ref-102] Johnstone TC, Nolan EM (2015). Beyond iron: non-classical biological functions of bacterial siderophores. Dalton Transaction.

[ref-103] Kabata-Pendias A (2000). Trace elements in soils and plants.

[ref-104] Kabata-Pendias A, Mukherjee AB, Arun B (2007). Trace elements from soil to human.

[ref-105] Kanwar MK, Bhardwaj R, Sparora PChowdhary, Sharma P, Kumar S (2013). Isolation and characterization of 24-Epibrassinolide from *Brassica juncea* L. and its effects on growth, Ni ion uptake, antioxidant defense of *Brassica* plants and in vitro cytotoxicity. Acta Physiologiae Plantarum.

[ref-106] Khan WU, Ahmad SR, Yasin NA, Ali A, Ahmad A, Akram W (2017). Application of *Bacillus megaterium* MCR-8 improved phytoextraction and stress alleviation of nickel in *Vinca rosea*. International Journal of Phytoremediation.

[ref-107] Khan N, Seshadri B, Bolan N, Saint C, Kirkham MB, Chowdhury S, Yamaguchi N, Lee DY, Li G, Kunhikrishnan A, Qi F, Karunanithi R, Qiu R, Zhu YG, Syu CH (2016). Root iron plaque on wetland plants as a dynamic pool of nutrients and contaminants. Advances in Agronomy.

[ref-108] Kim S, Takahashi M, Higuchi K, Tsunoda K, Nakanishi H, Yoshimura E, Mori S, Nishizawa NK (2005). Increased Ni cotianamine biosynthesis confers enhanced tolerance of high levels of metals, in particular nickel, to plants. Plant and Cell Physiology.

[ref-109] Kozlov MV (2005). Sources of variation in concentrations of nickel and copper in mountain birch foliage near a nickel-copper smelter at Monchegorsk, north-western Russia: results of long-term monitoring. Environmental Pollution.

[ref-110] Krämer U, Pickering IJ, Prince RC, Raskin I, Salt DE (2000). Subcellular localization and speciation of nickel in hyperaccumulator and non-accumulator Thlaspi species. Plant Physiology.

[ref-111] Kudoyarova GR, Kholodova VP, Veselov DS (2013). Current state of the problem of water relations in plants under water deficit. Russian Journal of Plant Physiology.

[ref-112] Kudoyarova GR, Melentiev AI, Martynenko EV, Timergalina LN, Arkhipova TN, Shendel GV, Kuz’mina LY, Dodd IC, Veselov SY (2014). Cytokinin producing bacteria stimulate amino acid deposition by wheat roots. Plant Physiology and Biochemistry.

[ref-113] Kuffner M, De Maria S, Puschenreiter M, Fallmann K, Wieshammer G, Gorfer M, Strauss J, Rivelli AR, Sessitsch A (2010). Culturable bacteria from Zn- and Cd accumulating *Salix caprea* with differentialeffects on plant growth and heavy metal availability. Journal of Applied Microbiology.

[ref-114] Kulac S, Cikili Y, Samet H, Filiz E (2018). Physiological, nutritional, and biochemical responses under nickel toxicity in black poplar (*Populus nigra*). Journal of BioScience and Biotechnology.

[ref-115] Kumar G, Kushwaha HR, Punjabi-Sabharwal V, Kumari S, Joshi R, Karan R, Mittal S, Singla-Pareek SL, Pareek A (2012). Clustered metallothionein genes are co-regulated in rice and ectopic expression of OsMT1e-P confers multiple abiotic stress tolerance in tobacco via ROS scavenging. BMC Plant Biology.

[ref-116] Kumar A, Verma JP (2018). Does plant—microbe interaction confer stress tolerance in plants: a review?. Microbiological Research.

[ref-117] Lebrette H, Borezée-Durant E, Martin L, Richaud P, Erba B, Cavazza C (2015). Novel insights into nickel import in *Staphylococcus aureus*: the positive role of free histidine and structural characterization of a new thiazolidine-type nickel chelator. Metallomics.

[ref-118] Lee S, Moon JS, Domier LL, Korban SS (2002). Molecular characterization of phytochelatin synthase expression in transgenic *Arabidopsis*. Plant Physiology and Biochemistry.

[ref-119] Lemaire S, Guillon B, Le Marechal P, Keryer E, Miginiac-Maslow M, Decottignies P (2004). New thioredoxin targets in the unicellular photosynthetic eukaryote *Chlamydomonas reinhardtii*. Proceedings of National Academy of Sciences of the United States of America.

[ref-120] Levy A, Gonzalez IS, Mittelviefhaus M, Clingenpeel S, Paredes SH, Miao J, Wang K, Devescovi G, Kyra K, Monteiro F, Alvarez BR, Lundberg DS, Lu T-Y, Lebeis S, Jin Z, McDonald M, Klein AP, Feltcher ME, Rio TG, Grant SR, Doty SL, E. Ley RE, Zhao B, Venturi V, Pelletier DA, Vorholt JA, Tringe SG, Woyke T, Dangl JL (2018). Genomic features of bacterial adaptation to plants. Nature Genetics.

[ref-121] Lhospice S, Gomez NO, Ouerdane L, Brutesco C, Ghssein G, Hajjar C, Liratni A, Wang S, Richaud P, Bleves S, Ball G, Borezée-Durant E, Lobinski R, Pignol D, Pascal Arnoux P, Voulhoux R (2017). Pseudomonas aeruginosa zinc uptake in chelating environment is primarily mediated by the metallophorepseudopaline. Scientific Reports.

[ref-122] Li Y-M, Chaney R, Brewer E, Roseberg R, Angle JS, Baker A, Reeves R, Nelkin J (2003). Development of a technology for commercial phytoextraction of nickel: economic and technical considerations. Plant and Soil.

[ref-123] Liesegang H, Lemke K, Siddiqui RA, Schlegel HG (1993). Characterization of the inducible nickel and cobalt resistance determinant cnr from pMOL28 of *Alcaligenes eutrophus* CH34. Journal of Bacteriology.

[ref-124] Liu F, Hewezi T, Lebeis SL, Pantalone V, Grewal PS, Staton ME (2019). Soil indigenous microbiome and plant genotypes cooperatively modify soybean rhizosphere microbiome assembly. BMC Microbiology.

[ref-125] Liu X, Hu P, Huang M, Tang Y, Li Y, Li L, Hou X (2016). The NF-YC-RGL2 module integrates GA and ABA signalling to regulate seed germination in Arabidopsis. Nature Communications.

[ref-126] Liu F, Xing S, Ma H, Du Z, Ma B (2013). Cytokinin-producing, plant growth-promoting rhizobacteria that confer resistance to drought stress in *Platycladus orientalis* container seedlings. Applied Microbiology and Biotechnology.

[ref-127] Llamas A, Sanz A (2008). Organ-distinctive changes in respiration rates of rice plants under nickel stress. Plant Growth Regulation.

[ref-128] Ma Y, Oliveira RS, Freitas H, Zhang C (2016a). Biochemical and molecular mechanisms of plant-microbe-metal interactions: relevance for phytoremediation. Frontiers in Plant Science.

[ref-129] Ma Y, Prasad MNV, Rajkumar M, Freitas H (2011a). Plant growth promoting rhizobacteria and endophytes accelerate phytoremediation of metalliferous soils. Biotechnology Advances.

[ref-130] Ma Y, Rajkumar M, Zhang CH, Freitas H (2016b). The beneficial role of bacterial endophytes in heavy metal phytoremediation. Journal of Environmental Management.

[ref-131] Ma Y, Rajkumar M, Freitas H (2009). Improvement of plant growth and nickel uptake by nickel resistant-plant-growth promoting bacteria. Journal of Hazardous Materials.

[ref-132] Ma Y, Rajkumar M, Luo YM, Freitas H (2011b). Inoculation of endophytic bacteriaon host and non-host plants e effects on plant growth and Ni uptake. Journal of Hazardous Materials.

[ref-133] Ma Y, Rajkumar M, Rocha I, Oliveira RS, Freitas H (2015). Serpentine bacteria influence metal translocation and bioconcentration of *Brassica juncea* and *Ricinus communis* grown in multi-metal polluted soils. Frontiers in Plant Science.

[ref-134] Maeda M, Hidaka M, Nakamura A, Masaki H, Uozumi T (1994). Cloning, sequencing, and expression of thermophilic *Bacillus* sp. strain TB-90 urease gene complex in *Escherichia coli*. Journal of Bacteriology.

[ref-135] Magaye R, Zhao J (2012). Recent progress in studies of metallic nickel and nickel-based nanoparticles’ genotoxicity and carcinogenicity. Environmental Toxicology and Pharmacology.

[ref-136] Mahmood S, Ishtiaq S, Yasin G, Irshad A (2016). Dose dependent rhizospheric Ni toxicity evaluation: Membrane stability and antioxidant potential of *Vigna species*. Chilean Journal of Agricultural Research.

[ref-137] Maitra S (2016). Study of genetic determinants of nickel and cadmium resistance in bacteria - a review. Journal of Current Microbiology and Applied Sciences.

[ref-138] Manna I, Bandyopadhyay M (2017). Engineered nickel oxide nanoparticle causes substantial physicochemical perturbation in plants. Frontiers in Chemistry.

[ref-139] Manoj SR, Karthik C, Kadirvelu K, Arulselvi PI, Shanmugasundaram T, Bruno B, Rajkumar M (2020). Understanding the molecular mechanisms for the enhanced phytoremediation of heavy metals through plant growth promoting rhizobacteria: a review. Journal of Environmental Management.

[ref-140] Mari S, Gendre D, Pianelli K, Ouerdane L, Lobinski R, Briat JF, Lebrun M, Czernic P (2006). Root - to shoot long-distance circulation of nicotianamine and nicotianamine-nickel chelates in the metal hyperaccumulator *Thlaspi caerulescens*. Journal of Experimental Botany.

[ref-141] Maruri-López I, Aviles-Baltazar NY, Buchala A, Serrano M (2019). Intra and extracellular journey of the phytohormone salicylic acid. Frontiers in Plant Science.

[ref-142] McMillan DJ, Mau M, Walker MJ (1998). Characterisation of the urease gene cluster in *Bordetella bronchiseptica*. Gene.

[ref-143] Mengoni A, Barzanti A, Gonnelli C, Gabrielli R, Bazzicalupo M (2001). Characterization of nickel-resistant bacteria isolated from serpentine soil. Environmental Microbiology.

[ref-144] Mergeay M, Nies D, Schlegel HG, Gerits J, Charles P, Van Gijsegem F (1985). *Alcaligenes eutrophus* CH34 is a facultative chemolithotroph with plasmid-bound resistance to heavy metals. Journal of Bacteriology.

[ref-145] Miethke M, Marahiel MA (2007). Siderophore-based iron acquisition and pathogen control. Microbiology and Molecular Biology Reviews.

[ref-146] Mikolay A, Nies DH (2009). The ABC transporter AtmA is involved in nickel and cobalt resistance of *Cupriavidusmetallidurans* strain CH34. Antonie Van Leeuwenhoek.

[ref-147] Minandri F, Imperi F, Frangipani E, Bonchi C, Visaggio D, Facchini M, Pasquali P, Bragonzi A, Visca P (2016). Role of iron uptake systems in *Pseudomonas aeruginosa* virulence and airway infection. Infection and Immunity.

[ref-148] Mirza N, Mahmood Q, Shah MM, Pervez A, Sultan S (2014). Plants as useful vectors to reduce environmental toxic arsenic content. Scientific World Journal.

[ref-149] Mittler R, Vanderauwera S, Gollery M, Van Breusegem F (2004). Reactive oxygen gene network of plants. Trends in Plant Science.

[ref-150] Mizuno T, Sonoda T, Horie K, Senoo K, Tanaka A, Mizuno N, Obata H (2003). Cloning and characterization of phytochelatin synthase from a nickel hyperaccumulator *Thlaspi japonicum* and its expression in yeast. Soil Science and Plant Nutrition.

[ref-151] Mulrooney SB, Hausinger RP (2003). Nickel uptake and utilization by microorganisms. FEMS Microbiology Reviews.

[ref-152] Najafi KS, Karimi N, Ghasempour H-R (2019). Salicylic acid and jasmonic acid restrains nickel toxicity by ameliorating antioxidant defense system in shoots of metallicolous and non-metallicolous *Alyssum inflatum* Náyr. populations. Plant Physiology and Biochemistry.

[ref-153] Nakazawa R, Ozawa T, Naito T, Kameda Y, Takenaga H (2001). Interactions between cadmium and nickel in phytochelatin biosynthesis and the detoxification of the two metals in suspensioncultured tobacco cells. Biologia Plantarum.

[ref-154] Naveed M, Bukhari SS, Mustafa A, Ditta A, Alamri S, El-Esawi MA, Rafique M, Ashraf S, Siddiqui MH (2020). Mitigation of nickel toxicity and growth promotion in sesame through the application of a bacterial endophyte and zeolite in nickel contaminated Soil. International Journal of Environmental Research and Public Health.

[ref-155] Naz I, Bano A, Ul-Hassan T (2009). Isolation of phytohormones producing plant growth promoting rhizobacteria from weeds growing in Khewra salt range, Pakistan and their implication in providing salt tolerance to *Glycine max* L. African Journal of Biotechnology.

[ref-156] Nedjimi B (2021). Phytoremediation: a sustainable environmental technology for heavy metals decontamination. SN Applied Sciences.

[ref-157] Nicks LJ, Chambers MF (1995). Farming for metals. Mining Environmental Management.

[ref-158] Nies DH, Nies A, Chu L, Silver S (1989). Expression and nucleotide sequence of a plasmid-determined divalent cation efflux system from *Alcaligeneseutrophus*. Proceedings of The National Academy of Sciences of the United States of America.

[ref-159] Nishida S, Aisu A, Mizuno T (2012). Induction of IRT1 by the nickel-induced iron-deficient response in *Arabidopsis*. Plant Signaling & Behavior.

[ref-160] Nishida S, Tsuzuki C, Kato A, Aisu A, Yoshida J, Mizuno T (2011). AtIRT1, the primary iron uptake transporter in the root, mediates excess nickel accumulation in *Arabidopsis thaliana*. Plant and Cell Physiology.

[ref-161] Noctor G, Reichhel J-P, Foyer CH (2018). ROS-related redox regulation and signaling in plants. Seminars in Cell & Developmental Biology.

[ref-162] Ortíz-Castro R, Contreras-Cornejo HA, Macías-Rodríguez L, López-Bucio J (2009). The role of microbial signals in plant growth and development. Plant Signaling and Behavior.

[ref-163] Pal KK, Tilak KVBR, Saxcna AK, Dey R, Singh CS (2001). Suppression of maize root diseases caused by *Macrophomina phaseolina*, Fusarium moniliforme and *Fusarium graminearum* by plant growth promoting rhizobacteria. Microbiological Research.

[ref-164] Pandey N, Sharma CP (2002). Effect of heavy metal Co^2+^, Ni^2+^ and Cd^2+^ on growth and metabolism of cabbage. Plant Science.

[ref-165] Panhwar QA, Jusop S, Naher UA, Othman R, Razi MI (2013). Application of potential phosphate-solubilizing bacteria and organic acids on phosphate solubilization from phosphate rock in aerobic rice. The Scientific World Journal.

[ref-166] Parida BK, Chhibba IM, Nayyar VK (2003). Influence of nickel contaminated soils on fenugreek (*Trigonella corniculata* L.) growth and mineral composition. Scientia Horticulturae.

[ref-167] Park JH, Bolan N, Megharaj M, Naidu R (2011). Isolation of phosphate solubilizing bacteria and their potential for lead immobilization in soil. Journal of Hazardous Materials.

[ref-168] Parlak KU (2016). Effect of nickel on growth and biochemical characteristics of wheat (*Triticumaestivum* L.) seedlings. NJAS - Wageningen Journal of Life Sciences.

[ref-169] Pasternak T, Groot EP, Kazantsev FV, Teale W, Omelyanchuk N, Kovrizhnykh V, Palme K, Mironova VV (2019). Salicylic acid affects root meristem patterning via auxin distribution in aconcentration-dependent manner. Plant Physiology.

[ref-170] Peeters K, Zuliani T, Žigon D, Milačič R, Ščančar J (2017). Nickel speciation in cocoa infusions using monolithic chromatography –Post-column ID-ICP-MS and Q-TOF-MS. Food Chemistry.

[ref-171] Pena LB, Zawoznik MS, Tomaro ML, Gallego SM (2008). Heavy metals effects on proteolytic system in sunflower leaves. Chemosphere.

[ref-172] Pérez-Palacios P, Romero-Aguilar A, Delgadillo J, Doukkali B, Caviedes MA, Rodríguez-Llorente ID, Pajuelo E (2017). Double genetically modified symbiotic system for improved Cu phytostabilization in legume roots. Environmental Science and Pollution Research.

[ref-173] Peroza EA, Freisinger E (2007). Metal ion binding properties of *Tricium aestivum* Ec-1 metallothionein: evidence supporting two separate metal thiolate clusters. Journal of Biological Inorganic Chemistry.

[ref-174] Persans MW, Nieman K, Salt DE (2001). Functional activity and role of cation-efflux family members in Ni hyperaccumulation in *Thlaspi goesingense*. Proceedings of the National Academy of Sciences of the United States of America.

[ref-175] Pietrini F, Iori V, Cheremisina A, Shevyakova NI, Radyukina N, Kuznetsov VV, Zacchini M (2015). Evaluation of nickel tolerance in *Amaranthus paniculatus* L. plants by measuring photosynthesis, oxidative status, antioxidative response and metal-binding molecule content. Environmental Science and Pollution Research.

[ref-176] Pishchik VN, Provorov NA, Vorobyov NI, Chizevskaya EP, Safronova VI, Kozhemyakov AP, Tuev AN (2009). Interactions between plants and associated bacteria in soils contaminated with heavy metals. Microbiology.

[ref-177] Pishchik VN, Vorob’ev NI, Provorov NA, KhomyakovYu V (2016). Mechanisms of plant and microbial adaptation to heavy metals in plant–microbial systems. Microbiology.

[ref-178] Pollard AJ, Reeves RD, Baker AJM (2014). Facultative hyperaccumulation of heavy metals and metalloids. Plant Science.

[ref-179] Prasad SM, Dwivedi R, Zeeshan M (2005). Growth, photosynthetic electron transport, and antioxidant responses of young soybean seedlings to simultaneous exposure of nickel and UV-B stress. Photosynthetica.

[ref-180] Qian J, Li D, Zhan G, Zhang L, Su W, Gao P (2012). Simultaneous biodegradation of Ni–citrate complexes and removalof nickel from solutions by *Pseudomonas alcaliphila*. Bioresource Technology.

[ref-181] Quiza L, St-Arnaud M, Yergeau E (2015). Harnessing phytomicrobiome signaling for rhizosphere microbiome engineering. Frontiers in Plant Science.

[ref-182] Rajindiran S, Dotaniya ML, Coumar MV, Panwar NR, Saha JK (2015). Heavy metal polluted soils in India: status and countermeasures. JNKVV Reseach Journal.

[ref-183] Rajkumar M, Ae N, Prasad MNV, Freitas H (2010). Potential of siderophore-producing bacteria for improving heavy metal phytoextraction. Trends in Biotechnology.

[ref-184] Rajkumar M, Ma Y, Freitas H (2013). Improvement of Ni phytostabilization by inoculation of Ni resistant *Bacillus megaterium* SR28C. Journal of Environmental Management.

[ref-185] Rajkumar M, Sandhya S, Prasad MNV, Freitas H (2012). Perspectives of plant associated microbes in heavy metal phytoremediation. Biotechnology Advances.

[ref-186] Rao KVM, Sresty TVS (2000). Antioxidative parameters in the seedlings of pigeonpea (Cajanuscajan (L.) Millspaugh) in response to Zn and Ni stresses. Plant Science.

[ref-187] Raza W, Shen Q (2020). Volatile organic compounds mediated plant-microbe interactions in soil. Molecular Aspects of Plant Beneficial Microbes in Agriculture, Chapter.

[ref-188] Reeves RD, Baker AJM, Raskin I, Ensley BD (2000). Metal-accumulating plants. Phytoremediation of toxic metals - using plants to clean up the environment.

[ref-189] Reis AR, Barcelos JPQ, Osório CRWS, Santos EF, Lisboa LAM, Santini JMK, Santos MJD, Junior EF, Campos M, Figueiredo PAM, Lavres J, Gratão PL (2017). A glimpse into the physiological, biochemical and nutritional status of soybean plants under Ni-stress conditions. Environmental and Experimental Botany.

[ref-190] Retamal-Morales G, Mehnert M, Schwabe R, Tischler D, Zapata C, Chávez R, Schlömann M, Levicán G (2018). Detection of arsenic-binding siderophores in arsenic-tolerating Actinobacteria by a modified CAS assay. Ecotoxicology and Environmental Safety.

[ref-191] Rizvi A, Bilal A, Zaidi A, Khan MS (2019). Heavy metal mediated phytotoxic impact on winter wheat: oxidative stress and microbial management of toxicity by *Bacillus subtilis* BM2. RSC Advances.

[ref-192] Rizwan M, Imtiaz M, Dai Z, Mehmood S, Adeel M, Liu J, Tu S (2017). Nickel stressed responses of rice in Ni subcellular distribution, antioxidant production, and osmolyte accumulation. Environmental Science and Pollution Research.

[ref-193] Rodrigue A, Effantin G, Berthelot MAM (2005). Identification of *rcnA* (*yohM*) a nickel and cobalt resistance gene in *Escherichia coli*. Journal of Bacteriology.

[ref-194] Roux A, Payne SM, Gilmore MS (2009). Microbial telesensing: probing the environment for friends, foes, and food. Cell Host and Microbe.

[ref-195] Roy M, McDonald LM (2015). Metal uptake in plants and health risk assessments in metal-contaminated smelter soils. Land Degradation and Development.

[ref-196] Sachan P, Lal N (2017). An overview of nickel (Ni^2+^) essentiality, toxicityand tolerance strategies in plants. Asian Journal of Biology.

[ref-197] Saif S, Khan MS (2018). Assessment of toxic impact of metals on proline, antioxidant enzymes, and biological characteristics of *Pseudomonas aeruginosa* inoculated *Cicer arietinum* grown in chromium and nickel-stressed sandy clay loam soils. Environmental Monitoring and Assessment.

[ref-198] Sarubbo LA, Rocha Jr R, Luna JM, Rufino RD, Santos VA, Banat IM (2015). Some aspects of heavy metals contamination remediation and role of biosurfactants. Chemistry and Ecology.

[ref-199] Schmidt T, Schlegel HG (1989). Nickel and cobalt resistance of various bacteria isolated from soil and highly polluted domestic and industrial wastes. FEMS Microbiology Ecology.

[ref-200] Schützendübel A, Polle A (2002). Plant responses to abiotic stresses: heavy metal-induced oxidative stress and protection by mycorrhization. Journal of Experimental Botany.

[ref-201] Seregin IV, Kozhevnikova AD (2006). Physiological role of nickel and its toxic effects on higher plants. Russian Journal of Plant Physiology.

[ref-202] Shahzad B, Tanveer M, Che Z, Rehman A, Cheema SASardarAlam, Sharmad A, Song H, Rehmane S, Zhaorong D (2018a). Role of 24-epibrassinolide (EBL) in mediating heavy metal and pesticide induced oxidative stress in plants: a review. Ecotoxicology and Environmental Safety.

[ref-203] Shahzad B, Tanveera M, Rehman A, Cheema SA, Fahad S, Rehman S, Sharma A (2018b). Nickel; whether toxic or essential for plants and environment - A review. Plant Physiology and Biochemistry.

[ref-204] Sharma SS, Dietz K-J (2009). The relationship between metal toxicity and cellular redox imbalance. Trends in Plant Science.

[ref-205] Sharma A, Sidhu GPS, Araniti F, Bali AS, Shahzad B, Tripathi DK, Brestic M, Skalicky M, Landi M (2020). The role of salicylic acid in plants exposed to heavy metals. Molecules.

[ref-206] Shukla D, Tiwari M, Tripathi RD, Nath P, Trivedi PK (2013). Synthetic phytochelatins complement a phytochelatin deficient *Arabidopsis* mutant and enhance the accumulation of heavy metal(loid)s. Biochemical and Biophysical Research Communications.

[ref-207] Siddiqui MH, Al-Whaibi MH, Ali HM, Sakran AM, Basalah MO, Al Khaishany MYY (2013). Mitigation of nickel stress by the exogenous application of salicylic acid and nitric oxide in wheat. Australian Journal of Crop Science.

[ref-208] Singh G, Agnihotri RK, Reshma RS, Ahmad M (2012). Effect of lead and nickel toxicity on chlorophyll and proline content of Urd (*Vigna mungo* L.) seedlings. International Journal of Plant Physiology and Biochemistry.

[ref-209] Singh AK, Cameotra SS (2013). Efficiency of lipopeptidebiosurfactants in removal of petroleum hydrocarbons and heavy metals from contaminated soil. Environmental Science and Pollution Research.

[ref-210] Sirhindi G, Mir MA, Abd-Allah EF, Ahmad P, Gucel S (2016). Jasmonic acid modulates the physio-biochemical attributes, antioxidant enzyme activity, and gene expression in glycine max under nickel toxicity. Frontiers in Plant Science.

[ref-211] Smith DL, Gravel V, Yergeau E (2017). Editorial: signaling in the phytomicrobiome. Frontiers in Plant Science.

[ref-212] Soares C, De Sousa A, Pinto A, Azenha M, Teixeira J, Azevedo AR, Fidalgo F (2016). Effect of 24-epibrassinolide on ROS content, antioxidant system, lipid peroxidation and Ni uptake in *Solanum nigrum* L. under Ni stress. Environmental and Experimental Botany.

[ref-213] Song W-Y, Mendoza-Cózatl DG, Lee Y, Schroeder JI, Ahn S-N, Lee H-S, Wicker T, Martinoia E (2014). Phytochelatinmetal (loid) transport into vacuoles shows different substrate preferences in barley and *Arabidopsis*. Plant, Cell and Environment.

[ref-214] Song P, Wen D, Guo ZX, Korakianitis T (2008). Oxidation investigation of nickel nanoparticles. Chemistry Chemical Physics.

[ref-215] Song Y, Zhang L, Li J, He X, Chen M, Deng Y (2018). High-potential accumulation and tolerance in the submerged hydrophyte *Hydrilla verticillata* (L.) *Royle* for nickel-contaminated water. Ecotoxicology and Environmental Safety.

[ref-216] Spence C, Bais H (2015). The role of plant growth regulators as chemical signals in plant–microbe interactions: a double edged sword. Current Opinion in Plant Biology.

[ref-217] Sreekanth T, Nagajyothi P, Lee K, Prasad T (2013). Occurrence, physiological responses and toxicity of nickel in plants. International Journal of Environmental Science and Technology.

[ref-218] Stahler FN, Odenbreit S, Haas R, Wilrich J, Van Vliet AH, Kusters JG, Kist M, Bereswill S (2006). The novel CznABC metal efflux pump is required for cadmium, zinc, and nickel resistance, urease modulation, and gastric colonization. Infection and Immunity.

[ref-219] Stearns J, Shah S, Greenbeerg B, Dixon DG, Glick BR (2005). Tolerance of transgenic canola expressing1-aminocyclopropane-1-carboxylic acid deaminase to growth inhibition by nickel. Plant Physiology and Biochemistry.

[ref-220] Stetsenko LA, Shevyakova NI, Kuznetsov VVRakitinVY (2011). Proline protects *Atropa belladonna* plants against nickel salt toxicity. Russian Journal of Plant Physiology.

[ref-221] Sujkowska-Rybkowska M, Kasowska D, Gediga K, Banasiewicz J, Stępkowski T (2020). Lotus corniculatus-rhizobia symbiosis under Ni, Co and Cr stress on ultramafic soil. Plant and Soil.

[ref-222] Taiz L, Zeiger E (2006). Plant Physiology.

[ref-223] Talebi M, Ebrahim B, Tabatabaei S, Akbarzadeh H (2019). Hyperaccumulation of Cu, Zn, Ni, and Cd in *Azolla* species inducing expression of methallothionein and phytochelatin synthase genes. Chemosphere.

[ref-224] Tank N, Saraf M (2009). Enhancement of plant growth and decontamination of nickel-spiked soil using PGPR. Journal of Basic Microbiology.

[ref-225] Thakur S, Sharma SS (2016). Characterization of seed germination, seedling growth, and associated metabolic responses of *Brassica juncea* L. cultivars to elevated nickel concentrations. Protoplasma.

[ref-226] Theriault G, Michael P, Nkongolo K (2016). Comprehensive transcriptome analysis of response to nickel stress in white birch (*Betula papyrifera*). PLOS ONE.

[ref-227] Tiwari S. Lata C (2018). Heavy metal stress, signaling, and tolerance due to plant-associated microbes: an overview. Frontiers in Plant Science.

[ref-228] Tsyganov VE, Tsyganova AV, Gorshkov AP, Seliverstova EV, Kim VE, Chizhevskaya EP, Belimov AA, Serova TA, Ivanova KA, Kulaeva OA, Kusakin PG, Kitaeva AB, Tikhonovich IA (2020). Efficacy of a plant-microbe system: *Pisum sativum* (L.) cadmium-tolerant mutant and *Rhizobium leguminosarum* strains, expressing pea metallothionein genes *PsMT1* and *PsMT2*, for cadmium phytoremediation. Frontiers in Microbiology.

[ref-229] Ude S, Arnold DL, Moon CD, Timms-Wilson T, Spiers AJ (2006). Biofilm formationand cellulose expression among diverse environmental Pseudomonas isolates. Environmental Microbiology.

[ref-230] Upadhyaya H, Panda SK, Bhattacharjee MK, Dutta S (2010). Role of arbuscular mycorrhiza in heavy metal tolerance in plants: prospects for phytoremidiation. Journal of Phytology.

[ref-231] Vandenkoornhuyse P, Quaiser A, Duhamel M, Le Van A, Dufresne A (2015). The importance of the microbiome of plant holobiont. New Phytologist.

[ref-232] Viehweger K (2014). How plants cope with heavy metals. Botanical Studies.

[ref-233] Visioli G, Gulli M, Marmiroli N (2014). Noccaeacaerulescens populations adapted to grow in metalliferous and nonmetalliferous soils: Ni tolerance, accumulation and expression analysis of genes involved in metal homeostasis. Environmental and Experimental Botany.

[ref-234] Vodyanitskii YN, Plekhanova IO, Prokopovich EV, Savichev AT (2011). Soil contamination with emissions of non-ferrous metallurgical plants. Eurasian Soil Science.

[ref-235] Wang H, Feng T, Peng X, Yan M, Tang X (2009). Up-regulation of chloroplastic antioxidant capacity is involved in alleviation of nickel toxicity of *Zea mays* L. by exogenous salicylic acid. Ecotoxicology and Environmental Safety.

[ref-236] Wani PA, Khan MS, Zaidi A (2008). Effect of heavy metal toxicity on growth, symbiosis, seed yield and metal uptake in pea grown in metal amended soil. Bull Environmental Contamination and Toxicology.

[ref-237] Wu X, Zhu ZB, Chen JH, Huang YF, Liu ZILi, Zou JW, Chen HY, Su NN, Cui J (2019). Transcriptome analysis revealed pivotal transporters involved in the reduction of cadmium accumulation in pakchoi (*Brassica chinensis* L.) by exogenous hydrogen-rich water. Chemosphere.

[ref-238] Yang XE, Baligar VC, Foster JC, Martens DC (1997). Accumulation and transport of nickel in relation to organic acids in ryegrass and maize grown with different nickel levels. In: Ando T, Fujita K, Mae T, Matsumoto H, Mori S, Sekiya J. (eds) Plant Nutrition for Sustainable Food Production and Environment. Developments in Plant and Soil Sciences.

[ref-239] Yu X, Ai C, Xin L, Zhou G (2011). The siderophore-producing bacterium, Bacillus subtilis CAS15, has a biocontrol effect on Fusarium wilt and promotes the growth of pepper. European Journal of Soil Biology.

[ref-240] Yusuf M, Fariduddin Q, Hayat S, Ahmad A (2011). Nickel: an overview of uptake, essentiality and toxicity in plants. Bulletin of Environmental Contamination and Toxicology.

[ref-241] Zahra S, Mahmood S, Noreen S, Akrem A (2018). Independent and combined nickel and cadmium induced lipid peroxidation of biological membranes and its mitigation through antioxidant enzymes in *Grewia asiatica* L. Pakistan Journal of Life and Social Sciences.

[ref-242] Zaid A, Mohammad FS, Wani SH, Siddique KMN (2019). Salicylic acid enhances nickel stress tolerance by up-regulating antioxidant defense and glyoxalase systems in mustard plants. Ecotoxicology and Environmental Safety.

[ref-243] Zaidi S, Usmani S, Singh BR, Musarrat J (2006). Significance of *Bacillus subtilis* strain SJ-101 as a bioinoculant for concurrent plant growth promotion and nickel accumulation in *Brassica juncea*. Chemosphere.

[ref-244] Zhang H, Zhang L, Li J, Chen M, An R-D (2020). Comparative study on the bioaccumulation of lead, cadmium and nickel and their toxic effects on the growth and enzyme defense strategies of a heavy metal accumulator, Hydrilla verticillata (L.f.) Royle. Environmental Science and Pollution Research.

[ref-245] Zhao J, Lu C, Tariq M, Xiao Q, Zhang W, Huang K, Lu Q, Lin K, Liu Z (2019). The response and tolerance mechanisms of lettuce (*Lactuca sativa* L.) exposed to nickel in a spiked soil system. Chemosphere.

[ref-246] Zhao Y, Xing L, Wang X, Hou Y-J, Gao J, Wang P, Duan CG, Zhu X, Zhu JK (2014). The ABA receptor PYL8 promotes lateral root growth by enhancing MYB77-dependent transcription of auxin-responsive genes. Science Signaling.

